# Post-marketing safety profile and clinical prioritization of adverse events with avacopan in ANCA-associated vasculitis: a FAERS pharmacovigilance analysis

**DOI:** 10.1080/0886022X.2025.2568971

**Published:** 2025-10-23

**Authors:** Zhenyao Yang, Chengzhi Wang, Shiju Wei, Yan Liu, Mingyang He, Chenglei Zheng, Wenjun Li, Dongdong Li, Peimin Liu

**Affiliations:** a The Second Clinical Medical College, Henan University of Chinese Medicine, Zhengzhou, Henan, China; b The First Clinical College, Henan University of Chinese Medicine, Zhengzhou, Henan, China; c The Second Affiliated Hospital, Henan University of Chinese Medicine, Zhengzhou, Henan, China

**Keywords:** Avacopan, ANCA-associated vasculitis, post-marketing safety, pharmacovigilance, FAERS, drug-induced liver injury

## Abstract

Avacopan, a novel C5a receptor inhibitor, offers therapeutic potential for ANCA-associated vasculitis. This study assessed its post-marketing safety using the FDA Adverse Event Reporting System (FAERS) from Q4 2021 to Q4 2024. Disproportionality analyses identified 75 adverse events (AEs) potentially linked to avacopan, with hepatobiliary disorders and infections being most prominent. A total of 62 AEs were classified as having moderate clinical priority, including drug-induced liver injury, renal failure, and deafness. Subgroup analysis revealed age- and sex-based differences in AE patterns. Logistic regression indicated that older age and concomitant use of rituximab or proton-pump inhibitors increased the risk of hepatobiliary AEs. These findings highlight the need for close monitoring of hepatic function and infection risk during avacopan therapy. Further research is warranted to explore the underlying mechanisms and potential drug interactions.

## Graphical abstract

**Figure:**
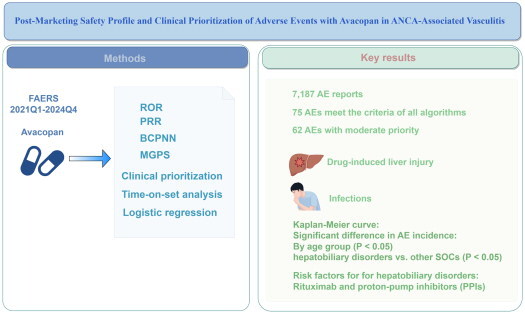

## 1. Introduction

ANCA-associated vasculitis (AAV) is a group of autoimmune diseases that primarily affect small blood vessels, often resulting in severe kidney damage and complications in other organs. Traditional treatment methods typically rely on the combination of corticosteroids and immunosuppressants; however, these approaches are often associated with significant side effects, such as an increased risk of infection and toxicity from long-term corticosteroid use, which severely impacts patients’ quality of life. Avacopan is a novel C5a receptor inhibitor that has demonstrated significant potential in the treatment of ANCA-associated vasculitis in recent years [1]. C5a is a potent inflammatory mediator that binds to the C5a receptor (C5aR), inducing the chemotaxis and activation of neutrophils, thereby exacerbating the inflammatory response. Avacopan competitively inhibits C5aR, reducing the activation and migration of neutrophils, which helps to alleviate the inflammatory response. In October 2021, the US Food and Drug Administration (FDA) approved avacopan for the treatment of ANCA-associated vasculitis, including granulomatosis with polyangiitis (GPA) and microscopic polyangiitis (MPA). Clinical trials have shown that when used in combination with standard treatment regimens, avacopan can achieve disease remission and improve kidney function in patients [2]. Furthermore, compared to traditional high-dose corticosteroid therapy, the use of avacopan does not increase the risk of adverse events and helps to reduce the side effects associated with corticosteroid use [3]. The unique mechanism of action of avacopan positions it as a groundbreaking treatment option, particularly in scenarios where reducing corticosteroid use is necessary. Although avacopan has not yet been approved as a primary treatment for AAV, it demonstrates significant potential as an adjunct therapy.

Although avacopan has demonstrated significant efficacy in clinical treatment, its expanding range of clinical applications has revealed associated adverse reactions. Current clinical observations indicate that the drug may cause a range of symptoms primarily related to gastrointestinal reactions, such as nausea, vomiting, and diarrhea, along with systemic reactions like headache, fatigue, and upper respiratory infections. These adverse reactions are mostly mild to moderate in nature and exhibit a time-dependent characteristic, with many symptoms gradually alleviating as treatment progresses [4]. Of particular concern is hepatotoxicity; numerous studies have reported liver toxicity associated with avacopan. For instance, a recent retrospective analysis from Japan indicated that elevated liver enzyme levels are the most common adverse reaction [5]. The US FDA has also added a warning to the label indicating that liver function tests should be conducted before initiating treatment with avacopan, and ongoing monitoring should occur during clinical use.

Due to the strict inclusion and exclusion criteria of clinical trials, the safety of avacopan in the general population may not be adequately represented, and some significant adverse events (AEs) may be overlooked. Importantly, there is currently a lack of real-world safety data regarding avacopan. Information on adverse drug events (ADEs) from spontaneous reporting system databases is a crucial resource for detecting signals of drug-related adverse reactions, and the FAERS provides a platform for ADE analysis. Therefore, this study employs data mining to conduct a post-marketing evaluation of avacopan, aiming to provide more comprehensive and reliable results.

## 2. Materials and methods

### 2.1. Data source

This study is an observational and retrospective pharmacovigilance analysis of the FDA Adverse Event Reporting System (FAERS) database, utilizing data from Q4 2021 to Q4 2024, accessed and downloaded from the FAERS (FAERS Quarterly Data Extract Files). Each file contains seven types of data, including DEMO (patient demographics), REAC (all terms coded for events from the Medical Dictionary for Regulatory Activities, MedDRA), DRUG (drug/biologic information), OUTC (patient outcomes), RPSR (report source), THER (treatment start and end dates), and INDI (indications for drug use). In FAERS, reporters classify exposure to specific drugs as primary suspect (PS), secondary suspect (SS), concomitant (C), or interaction (I).

### 2.2. Data extraction and identification

We followed the Medical Dictionary for Regulatory Activities (MedDRA version 25.0). MedDRA includes five levels of results: System Organ Class (SOC), High-Level Group Term (HLGT), High-Level Term (HLT), Preferred Term (PT), and Lowest Level Term (LLT). Following FDA recommendations, we removed duplicate data. If cases had the same CASEID, we retained the report with the most recent FDA_DT; if both CASEID and FDA_DT were the same, we retained the report with the larger PRIMARYID. After removing duplicates, we still found some duplicate PRIMARYIDs, necessitating a secondary round of duplicate removal. We identified 5,533,239 patients in FAERS (DEMO table), retaining 4,820,226 patients after removing duplicates. To avoid potential misclassification of AEs and enhance the accuracy of our analysis, we focused on cases designated as primary suspect (PS) drugs. Ultimately, we included 3,150 reports where avacopan was the primary treatment and 7,187 adverse events for further analysis.

### 2.3. Disproportionality analysis

Disproportionality analysis is a comprehensive approach that formulates hypotheses regarding potential drug-event associations by comparing the reporting rates of specific AEs for a single drug or pharmacological class with the reporting rates of the same AEs in a reference group, this method is currently one of the more established techniques for detecting AE signals [6,7]. We applied multiple disproportionality analysis methods, including Reporting Odds Ratio (ROR), Proportional Reporting Ratio (PRR), Bayesian Confidence Propagation Neural Network (BCPNN), and Multi-item Gamma Poisson Shrinker (MGPS). In pharmacovigilance and large-scale spontaneous reporting databases, ROR was selected as the primary method due to its widespread application and proven effectiveness [8]. The choice of PRR as an analytical tool is based on its stability in handling small sample sizes and the analysis of rare AEs, which aligns with the standard methodological approach recommended by the FDA [9]. The implementation of BCPNN aims to address the issue of random errors in small samples and provides reliable estimates of signal strength through the Information Criterion (IC) [10]. MGPS is particularly valuable for analyzing combinations of multiple drug events; it employs Bayesian shrinkage to reduce false positive results and is considered a more rigorous algorithm [11]. To minimize bias, reduce the false positive rate, and strengthen the detection threshold, we required that all four methods meet the criteria simultaneously to confirm a valid signal. Notably, we need to interpret events with counts less than 10 with caution. We implemented several well-validated criteria across different methods, with specific calculation standards listed in Supplementary Table S1. To address potential confounding factors and biases, we conducted stratified analyses based on age and sex; however, weight data were insufficient for analysis.

### 2.4. Classification and prioritization of relevant disproportionality signals

In this study, we utilized previously described methods to assess clinical priorities [12], for avacopan, AEs that showed positive signals in at least one disproportionality analysis were ranked based on a semi-quantitative scoring system evaluating the following criteria (Table 1).

**Table 1.** Basic information on the AE reports related to avacopan.

Characteristics	Reports, *n* (%)
Sex	
Female	1,195 (37.9%)
Male	827 (26.3%)
Unknown	1,128 (35.8%)
Weight (kg)	
<50	62 (2.0%)
50–100	196 (6.2%)
≥100	24 (0.8%)
Unknown	2,868 (91.0%)
Age (years)	
<18	42 (1.3%)
18–65	908 (28.8%)
65–85	820 (26.0%)
≥85	54 (1.7%)
Unknown	1,326 (42.1%)
Reporter	
Consumer	1,637 (52.0%)
Physician	685 (21.7%)
Other health-professional	398 (12.6%)
Lawyer	529 (3.3%)
Pharmacist	176 (5.6%)
Unknown	254 (8.1%)
Reported countries	
United States	2,403 (76.3%)
Japan	399 (12.7%)
United Kingdom	101 (3.2%)
Canada	94 (3.0%)
France	48 (1.5%)
Germany	30 (1.0%)
Other	75 (2.4%)
Outcomes	
Hospitalization	734 (23.3%)
Other Serious	559 (17.7%)
Death	237 (7.5%)
Life Threatening	34 (1.1%)
Disability	3 (0.1%)
Required intervention to prevent permanent impairment/damage	1 (0.0%)
Unknown	1,582 (50.2%)
Year	
2021	1 (0.1%)
2022	360 (11.4%)
2023	1,547 (49.1%)
2024	1,242 (39.4%)
Time-to-onset (days)	
0–30	202 (35.9%)
31–60	119 (21.2%)
61–90	58 (10.3%)
91–120	36 (6.4%)
121–150	16 (2.8%)
151–180	19 (3.4%)
181–360	68 (12.1%)
>360	44 (7.8%)

1. Clinical Relevance: We utilized the lists of ‘important medical events’ (IMEs—serious events—version 2.6.0) and ‘designated medical events’ (DMEs—rare but serious events likely to be drug-induced) provided by the European Medicines Agency [13].
2. Reporting Rate: The ratio of adverse events of interest to other adverse events (i.e., the ratio of cases to non-cases), to align with clinical trial reporting, the following traditional categories were used: very common (≥10%), common (1–10%), and uncommon (≤1%).
3. Signal Stability: The consistency/robustness of disproportional signals across multiple analyses. The highest score is awarded for complete consistency, which is determined by the disproportional signals in four analyses using different comparators.
4. Reported Case Fatality Rate: The ratio of reports of death to all adverse events. Death is generally considered an important medical event, closely related to both the medication and the patient’s underlying health condition. For these reasons, we adopted a conservative approach, awarding the highest score only when the case fatality rate exceeds 50%.

Scores of 0–2, 3–5, and 6–8 were respectively assigned to AEs classified as low, medium, and high priority.

### 2.5. Cumulative incidence and time‐to‐onset

Time-to-onset (TTO) is defined as the time from the initiation of drug treatment to the occurrence of an adverse reaction. Therefore, we only analyzed reports that contained onset-time data. We used the Kaplan-Meier method to plot the cumulative incidence of adverse reactions for avacopan and employed the log-rank test to compare the cumulative incidence of adverse reactions between age subgroups, sex subgroups, and hepatobiliary disorders versus other SOC levels. TTO was described using the median and interquartile range (IQR).

### 2.6. Statistical analysis

We conducted statistical analyses using RStudio (version 4.3.2). Descriptive statistical analyses were performed on the target cases, focusing on variables such as sex, age, outcomes, and the timing of adverse reactions. Hepatotoxicity induced by avacopan is often a reason for treatment discontinuation; therefore, we selected AEs under the SOC level of hepatobiliary disorders as outcomes for univariate and multivariate logistic regression analyses to determine the odds ratios (ORs) for avacopan-induced hepatotoxicity across different exposure factors such as sex, age, and commonly used concomitant medications. The *p*-value of <0.05 was considered statistically significant.

## 3. Results

### 3.1. Basic characteristics of avacopan-related ADEs

**Figure 1.**
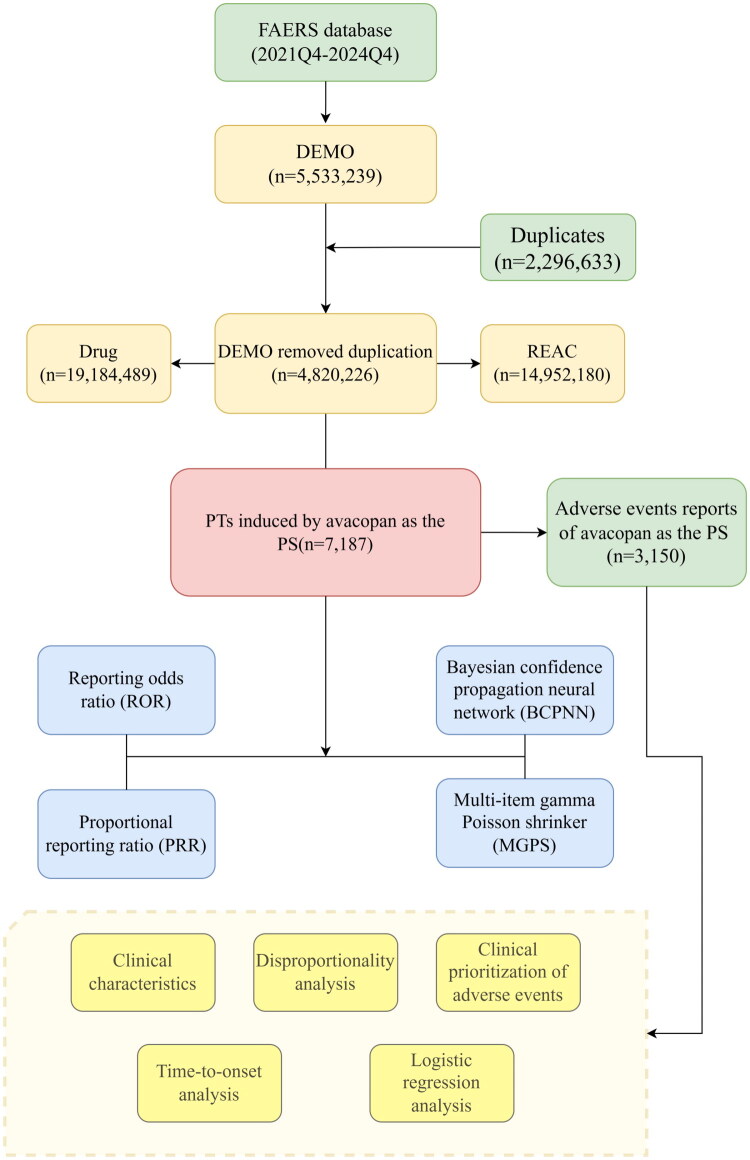
Flowchart of the comprehensive analysis of avacopan-related adverse reactions from the FAERS database.

The entire research process is illustrated in Figure 1. From 2021 to 2024, a total of 14,952,180 adverse event reports were collected from the FAERS database. Among these, 7,187 reports identified avacopan as the primary suspected drug in ADEs. In these reports, the proportion of females (37.9%) was higher than that of males (26.3%). Regarding age distribution, patients over 65 years old accounted for the majority of individual case safety reports (26.0%), excluding those with unknown age. The majority of reports (52.0%) were submitted by consumers. The United States had the highest number of reports (76.3%). In terms of clinical outcomes, the most common serious ADE, excluding those with unspecified severity, was hospitalization (23.2%), followed by death (7.5%). In the temporal distribution of ADEs, excluding cases with unknown duration of administration, the highest proportion of adverse reactions occurred within 30 days, accounting for 35.9%. The incidence of AEs decreased over time; for more detailed information, please refer to Table 2. Between days 31 and 60, 119 AEs (21.2%) were reported, while between days 61 and 90, 58 AEs (10.3%) were reported. Notably, our data indicate that 32.6% of patients may still experience AEs after receiving avacopan treatment for ≥90 days. These findings highlight the importance of ongoing monitoring for potential AEs throughout the duration of avacopan treatment. Supplementary Table S3 lists the top 10 AEs at the PT level associated with hospitalization and death. The most common AE associated with death was pneumonia (14 occurrences), followed by COVID-19 (13 occurrences) and infection (9 occurrences). The most common AE associated with life-threatening events was pneumonia (4 occurrences), followed by vanishing bile duct syndrome (4 occurrences) and cerebrovascular accident (9 occurrences). The top three AEs related to hospitalization were pneumonia (33 occurrences), nausea (25 occurrences), and COVID-19 (23 occurrences).

**Table 2.** The signal strength of AEs of avacopan at the SOC level.

SOC	*N*	ROR (95%Cl)	PRR (χ²)	EBGM (EBGM05)	IC (IC025)
Surgical and medical procedures#	578	5.39 (4.95–5.87)	5.03 (1,894.55)	5.02 (4.68)	2.33 (2.2)
Investigations	469	1.13 (1.03–1.24)	1.12 (6.4)	1.12 (1.04)	0.16 (0.03)
General disorders and administration site conditions	1,194	0.92 (0.86–0.98)	0.93 (7.45)	0.93 (0.88)	−0.1 (−0.19)
Vascular disorders	192	1.48 (1.28–1.71)	1.47 (29)	1.47 (1.3)	0.55 (0.34)
Skin and subcutaneous tissue disorders	315	0.84 (0.75–0.94)	0.85 (9.19)	0.85 (0.77)	−0.24 (−0.41)
Nervous system disorders	499	0.97 (0.89–1.06)	0.97 (0.37)	0.97 (0.9)	−0.04 (−0.17)
Respiratory, thoracic and mediastinal disorders	363	1.13 (1.01–1.25)	1.12 (4.8)	1.12 (1.02)	0.16 (0.01)
Infections and infestations	712	1.73 (1.6–1.87)	1.66 (199.22)	1.66 (1.56)	0.73 (0.62)
Hepatobiliary disorders#	296	5.05 (4.49–5.67)	4.88 (918.77)	4.87 (4.42)	2.28 (2.11)
Gastrointestinal disorders	840	1.55 (1.44–1.66)	1.48 (143.52)	1.48 (1.4)	0.57 (0.46)
Cardiac disorders	87	0.65 (0.52–0.8)	0.65 (16.68)	0.65 (0.54)	−0.62 (−0.93)
Injury, poisoning and procedural complications	607	0.6 (0.55–0.65)	0.63 (149.4)	0.63 (0.59)	−0.66 (−0.78)
Psychiatric disorders	136	0.35 (0.3–0.42)	0.37 (157.33)	0.37 (0.32)	−1.45 (−1.7)
Metabolism and nutrition disorders	129	0.94 (0.79–1.12)	0.94 (0.49)	0.94 (0.81)	−0.09 (−0.34)
Product issues	17	0.11 (0.07–0.18)	0.12 (116.47)	0.12 (0.08)	−3.1 (−3.78)
Musculoskeletal and connective tissue disorders	210	0.55 (0.48–0.63)	0.56 (74.16)	0.57 (0.5)	−0.82 (−1.02)
Eye disorders	82	0.57 (0.45–0.7)	0.57 (27.11)	0.57 (0.48)	−0.81 (−1.13)
Endocrine disorders	4	0.2 (0.08–0.54)	0.2 (12.69)	0.2 (0.09)	−2.31 (−3.6)
Renal and urinary disorders	198	1.69 (1.46–1.94)	1.67 (53.76)	1.67 (1.48)	0.74 (0.53)
Ear and labyrinth disorders	38	1.33 (0.96–1.82)	1.32 (3.03)	1.32 (1.01)	0.41 (−0.06)
Neoplasms benign, malignant and unspecified (incl cysts and polyps)	31	0.14 (0.1–0.19)	0.14 (168.4)	0.14 (0.1)	−2.83 (−3.34)
Immune system disorders	72	0.89 (0.7–1.12)	0.89 (1)	0.89 (0.73)	−0.17 (−0.51)
Blood and lymphatic system disorders	100	0.78 (0.64–0.95)	0.78 (6.13)	0.78 (0.66)	−0.35 (−0.64)
Reproductive system and breast disorders	7	0.17 (0.08–0.35)	0.17 (28.69)	0.17 (0.09)	−2.56 (−3.58)
Social circumstances	9	0.27 (0.14–0.52)	0.27 (17.7)	0.27 (0.16)	−1.88 (−2.8)
Congenital, familial and genetic disorders	2	0.11 (0.03–0.43)	0.11 (14.89)	0.11 (0.03)	−3.22 (−4.89)

*Note: N* < 10, to be interpreted with caution; The asterisk (#) means statistically significant association, the adverse events are detected as signals.

### 3.2. Signal detection for avacopan

In this study, the analysis of adverse event reports associated with avacopan revealed that the drug-related adverse reactions involved 24 different SOCs. Statistically, the SOCs that met all four signal detection criteria and were significantly associated with avacopan-related AEs were hepatobiliary disorders (*N* = 296, ROR 5.05, PRR 4.88, IC 2.28, EBGM 4.87) and various surgical and medical procedures (*N* = 578, ROR 5.39, PRR 5.03, IC 2.33, EBGM 5.02). For more details, please refer to Table 2.

**Figure 2.**
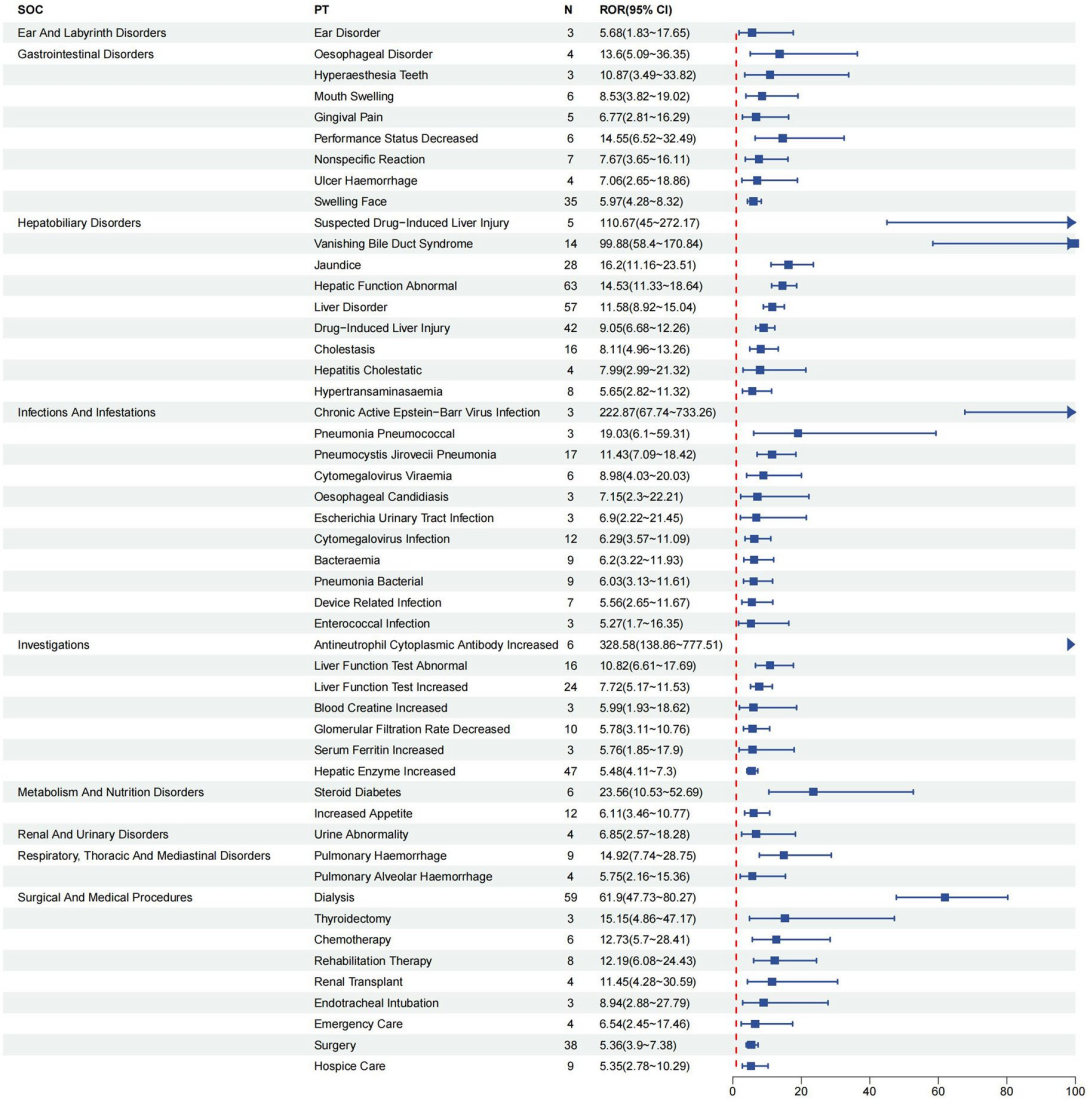
The top 50 signal strength of avacopan-related AEs sorted by the ROR algorithm at the PT level. *Note: N* < 10, to be interpreted with caution.

At the PT level, four algorithms were used to analyze drug adverse reactions and assess their compliance with various screening criteria. 75 AEs met the criteria of the four algorithms, and the results were categorized according to SOC, as shown in Table 3. The study results showed that certain AEs had high signal strength, such as antineutrophil cytoplasmic antibody increased (*N* = 6, ROR 328.58, PRR 328.30, IC 8.15, EBGM 283.67), chronic active Epstein-Barr virus infection (*N* = 3, ROR 222.87, PRR 222.78, IC 7.65, EBGM 201.31), and suspected drug-induced liver injury (*N* = 5, ROR 110.67, PRR 110.6, IC 1.22, EBGM 2.33). The most commonly reported AEs were diarrhea (*N* = 180, ROR 2.37, PRR 2.33, IC 6.16, EBGM 71.67), abdominal discomfort (*N* = 74, ROR 3.61, PRR 3.58, IC 1.84, EBGM 3.58), illness (*N* = 70, ROR 2.5, PRR 2.48, IC 1.31, EBGM 2.48), hepatic function abnormal (*N* = 63, ROR 14.53, PRR 14.41, IC 3.84, EBGM 14.32), and hypertension (*N* = 62, ROR 2.63, PRR 2.62, IC 1.39, EBGM 2.62). We also filtered the results using the most commonly used ROR algorithm and plotted a forest plot of the top 50 by signal strength, as detailed in Figure 2. We found that AEs related to hepatobiliary disorders and infections had high signal strengths; specifically, vanishing bile duct syndrome, pulmonary hemorrhage, and esophageal disorder, although reported infrequently, showed high signal strength and warrant our attention.

**Table 3.** Classification of AEs meeting four algorithms at the SOC level.

SOC	PT	*N*	ROR (95%Cl)	PRR (χ²)	EBGM (EBGM05)	IC (IC025)
Ear And Labyrinth Disorders	Deafness	10	3.52 (1.89–6.55)	3.52 (18.01)	3.52 (2.09)	1.81 (0.94)
	Ear Disorder	3	5.68 (1.83–17.65)	5.68 (11.54)	5.67 (2.2)	2.5 (1.06)
Gastrointestinal Disorders	Diarrhea	180	2.37 (2.04–2.74)	2.33 (138.41)	2.33 (2.06)	1.22 (1)
	Abdominal Discomfort	74	3.61 (2.87–4.54)	3.58 (137.76)	3.58 (2.95)	1.84 (1.5)
	Mouth Swelling	6	8.53 (3.82–19.02)	8.52 (39.67)	8.49 (4.34)	3.09 (1.99)
	Gingival Pain	5	6.77 (2.81–16.29)	6.76 (24.48)	6.75 (3.23)	2.75 (1.57)
	Esophageal Disorder	4	13.6 (5.09–36.35)	13.59 (46.35)	13.51 (5.93)	3.76 (2.46)
	Hyperaesthesia Teeth	3	10.87 (3.49–33.82)	10.87 (26.74)	10.82 (4.18)	3.44 (1.99)
General Disorders And Administration Site Conditions	Illness	70	2.5 (1.97–3.16)	2.48 (62.08)	2.48 (2.04)	1.31 (0.97)
	Swelling Face	35	5.97 (4.28–8.32)	5.94 (143.61)	5.93 (4.49)	2.57 (2.08)
	Nonspecific Reaction	7	7.67 (3.65–16.11)	7.66 (40.39)	7.64 (4.1)	2.93 (1.91)
	Performance Status Decreased	6	14.55 (6.52–32.49)	14.54 (75.13)	14.45 (7.38)	3.85 (2.76)
	Ulcer Hemorrhage	4	7.06 (2.65–18.86)	7.06 (20.74)	7.04 (3.1)	2.82 (1.52)
Hepatobiliary Disorders	Hepatic Function Abnormal	63	14.53 (11.33–18.64)	14.41 (781.5)	14.32 (11.63)	3.84 (3.48)
	Liver Disorder	57	11.58 (8.92–15.04)	11.49 (543.51)	11.44 (9.19)	3.52 (3.13)
	Drug-Induced Liver Injury	42	9.05 (6.68–12.26)	9 (297.62)	8.97 (6.95)	3.16 (2.72)
	Jaundice	28	16.2 (11.16–23.51)	16.14 (394.58)	16.02 (11.73)	4 (3.46)
	Cholestasis	16	8.11 (4.96–13.26)	8.09 (99.13)	8.07 (5.35)	3.01 (2.31)
	Vanishing Bile Duct Syndrome	14	99.88 (58.4–170.84)	99.69 (1,305.25)	95.17 (60.74)	6.57 (5.81)
	Hypertransaminasaemia	8	5.65 (2.82–11.32)	5.65 (30.51)	5.63 (3.15)	2.49 (1.53)
	Suspected Drug-Induced Liver Injury	5	110.67 (45 − 272.17)	110.6 (515.61)	105.06 (49.48)	6.72 (5.5)
	Hepatitis Cholestatic	4	7.99 (2.99–21.32)	7.98 (24.34)	7.96 (3.5)	2.99 (1.7)
	Hepatitis Acute	3	5.23 (1.68–16.23)	5.22 (10.22)	5.21 (2.02)	2.38 (0.94)
Immune System Disorders	Immunosuppression	5	4.25 (1.77–10.21)	4.24 (12.37)	4.24 (2.03)	2.08 (0.9)
Infections And Infestations	Infection	55	3.01 (2.31–3.93)	3 (73.3)	2.99 (2.4)	1.58 (1.2)
	Lower Respiratory Tract Infection	20	3.36 (2.17–5.22)	3.36 (33.07)	3.35 (2.32)	1.75 (1.11)
	*Pneumocystis Jirovecii* Pneumonia	17	11.43 (7.09–18.42)	11.41 (160.55)	11.35 (7.61)	3.5 (2.82)
	Cytomegalovirus Infection	12	6.29 (3.57–11.09)	6.28 (53.16)	6.27 (3.9)	2.65 (1.85)
	Bacteraemia	9	6.2 (3.22–11.93)	6.19 (39.06)	6.17 (3.57)	2.63 (1.71)
	Pneumonia Bacterial	9	6.03 (3.13–11.61)	6.03 (37.63)	6.01 (3.48)	2.59 (1.67)
	Device Related Infection	7	5.56 (2.65–11.67)	5.55 (26.07)	5.54 (2.98)	2.47 (1.45)
	Cytomegalovirus Viraemia	6	8.98 (4.03–20.03)	8.98 (42.34)	8.94 (4.57)	3.16 (2.07)
	Esophageal Candidiasis	3	7.15 (2.3–22.21)	7.15 (15.8)	7.12 (2.76)	2.83 (1.39)
	Enterococcal Infection	3	5.27 (1.7–16.35)	5.26 (10.34)	5.25 (2.04)	2.39 (0.95)
	Escherichia Urinary Tract Infection	3	6.9 (2.22–21.45)	6.9 (15.09)	6.88 (2.66)	2.78 (1.34)
	Chronic Active Epstein-Barr Virus Infection	3	222.87 (67.74–733.26)	222.78 (598.25)	201.31 (74.32)	7.65 (6.12)
	Pneumonia Pneumococcal	3	19.03 (6.1–59.31)	19.02 (50.75)	18.85 (7.28)	4.24 (2.79)
Investigations	Hepatic Enzyme Increased	47	5.48 (4.11–7.3)	5.45 (170.47)	5.44 (4.28)	2.44 (2.02)
	Alanine Aminotransferase Increased	25	4.76 (3.21–7.05)	4.74 (73.71)	4.73 (3.41)	2.24 (1.68)
	Liver Function Test Increased	24	7.72 (5.17–11.53)	7.7 (139.37)	7.67 (5.48)	2.94 (2.36)
	Blood Creatinine Increased	23	3.53 (2.34–5.31)	3.52 (41.41)	3.51 (2.49)	1.81 (1.22)
	Aspartate Aminotransferase Increased	22	4.91 (3.23–7.47)	4.9 (68.2)	4.89 (3.45)	2.29 (1.69)
	Liver Function Test Abnormal	16	10.82 (6.61–17.69)	10.79 (141.49)	10.74 (7.12)	3.43 (2.72)
	Blood Pressure Abnormal	13	4.84 (2.8–8.34)	4.83 (39.39)	4.82 (3.06)	2.27 (1.5)
	Glomerular Filtration Rate Decreased	10	5.78 (3.11–10.76)	5.78 (39.39)	5.76 (3.43)	2.53 (1.65)
	Blood Alkaline Phosphatase Increased	7	4.22 (2.01–8.86)	4.22 (17.14)	4.21 (2.26)	2.07 (1.05)
	Antineutrophil Cytoplasmic Antibody Increased	6	328.58 (138.86–777.51)	328.3 (1,690.86)	283.67 (137.98)	8.15 (6.98)
	Inflammatory Marker Increased	4	4.9 (1.84–13.08)	4.9 (12.38)	4.89 (2.15)	2.29 (1)
	Serum Ferritin Increased	3	5.76 (1.85–17.9)	5.76 (11.77)	5.75 (2.23)	2.52 (1.08)
	Blood Creatinine Increased	3	5.99 (1.93–18.62)	5.99 (12.44)	5.98 (2.32)	2.58 (1.13)
Metabolism And Nutrition Disorders	Increased Appetite	12	6.11 (3.46–10.77)	6.1 (51)	6.08 (3.78)	2.6 (1.8)
	Steroid Diabetes	6	23.56 (10.53–52.69)	23.54 (128.04)	23.29 (11.87)	4.54 (3.44)
Renal And Urinary Disorders	Renal Impairment	38	3.71 (2.69–5.1)	3.69 (74.61)	3.69 (2.82)	1.88 (1.42)
	Renal Disorder	26	5.23 (3.56–7.7)	5.22 (88.5)	5.21 (3.77)	2.38 (1.82)
	Proteinuria	9	3.82 (1.98–7.34)	3.81 (18.65)	3.81 (2.2)	1.93 (1.01)
	End Stage Renal Disease	7	4.16 (1.98–8.74)	4.16 (16.78)	4.15 (2.23)	2.05 (1.03)
	Urine Abnormality	4	6.85 (2.57–18.28)	6.85 (19.9)	6.83 (3)	2.77 (1.48)
Respiratory, Thoracic And Mediastinal Disorders	Lung Disorder	18	3.21 (2.02–5.11)	3.21 (27.35)	3.21 (2.18)	1.68 (1.02)
	Pulmonary Hemorrhage	9	14.92 (7.74–28.75)	14.9 (115.88)	14.8 (8.55)	3.89 (2.97)
	Pharyngeal Swelling	8	3.82 (1.91–7.66)	3.82 (16.64)	3.82 (2.14)	1.93 (0.97)
	Pulmonary Alveolar Hemorrhage	4	5.75 (2.16–15.36)	5.75 (15.66)	5.74 (2.52)	2.52 (1.23)
Skin And Subcutaneous Tissue Disorders	Alopecia	50	2.56 (1.94–3.38)	2.55 (47.07)	2.55 (2.02)	1.35 (0.94)
Surgical And Medical Procedures	Dialysis	59	61.9 (47.73–80.27)	61.4 (3,405.44)	59.67 (48)	5.9 (5.52)
	Surgery	38	5.36 (3.9–7.38)	5.34 (133.74)	5.33 (4.08)	2.41 (1.95)
	Therapy Cessation	25	4 (2.7–5.93)	3.99 (55.94)	3.98 (2.87)	1.99 (1.43)
	Hospice Care	9	5.35 (2.78–10.29)	5.34 (31.68)	5.33 (3.08)	2.41 (1.5)
	Rehabilitation Therapy	8	12.19 (6.08–24.43)	12.18 (81.6)	12.11 (6.77)	3.6 (2.63)
	Chemotherapy	6	12.73 (5.7–28.41)	12.72 (64.39)	12.65 (6.46)	3.66 (2.57)
	Cardiac Operation	5	5.24 (2.18–12.61)	5.24 (17.1)	5.23 (2.51)	2.39 (1.21)
	Renal Transplant	4	11.45 (4.28–30.59)	11.44 (37.9)	11.38 (5)	3.51 (2.21)
	Emergency Care	4	6.54 (2.45–17.46)	6.54 (18.71)	6.52 (2.87)	2.71 (1.41)
	Endotracheal Intubation	3	8.94 (2.88–27.79)	8.94 (21.06)	8.9 (3.45)	3.15 (1.71)
	Thyroidectomy	3	15.15 (4.86–47.17)	15.14 (39.33)	15.04 (5.81)	3.91 (2.46)
Vascular Disorders	Hypertension	62	2.63 (2.05–3.38)	2.62 (62.18)	2.62 (2.12)	1.39 (1.02)
	Deep Vein Thrombosis	14	3.62 (2.14–6.13)	3.62 (26.51)	3.61 (2.33)	1.85 (1.11)

*Note: N* < 10, to be interpreted with caution.

### 3.3. Subgroup analysis

**Figure 3.**
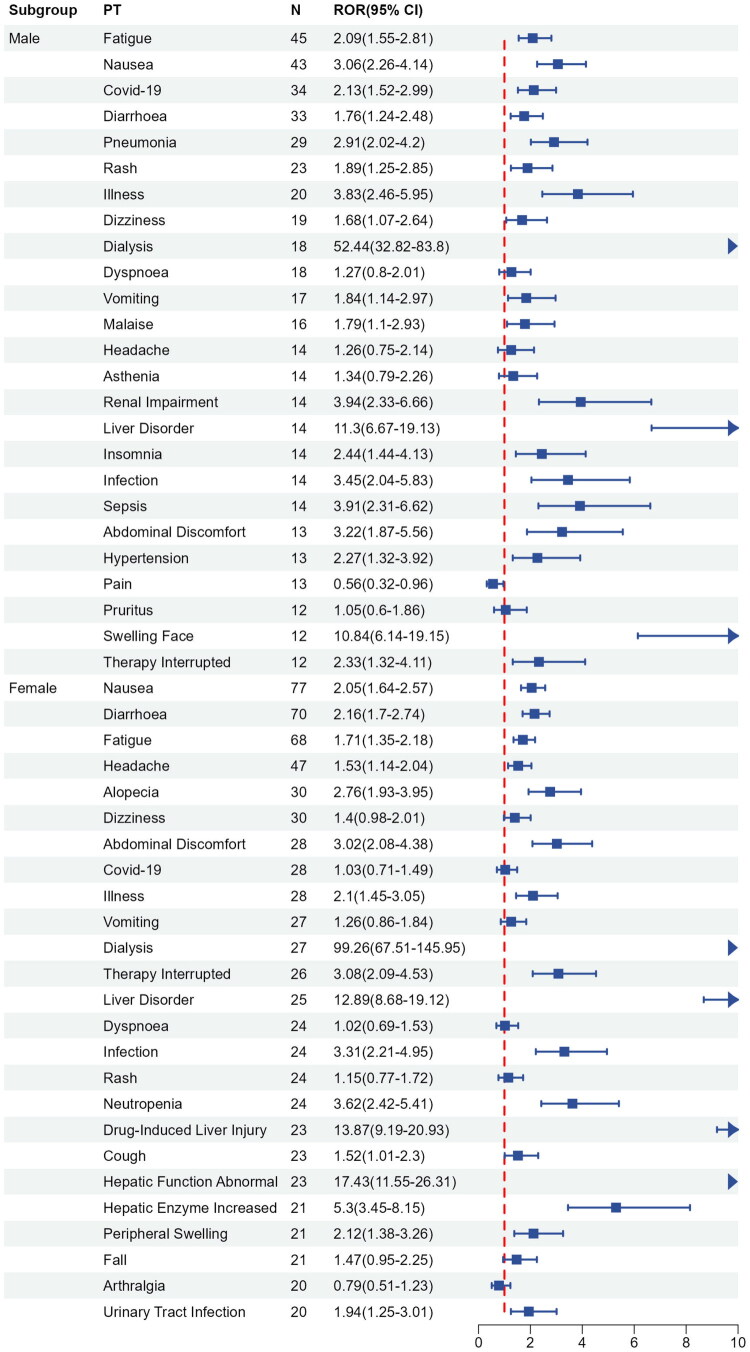
Sex-based subgroup analysis of avacopan-related AEs. *Note: N* < 10, to be interpreted with caution.

Subsequently, we conducted subgroup analyses to mitigate the potential confounding effects of demographic characteristics on the outcomes. In the male subgroup, fatigue was the most common event, followed by nausea, COVID-19, diarrhea, and pneumonia. Further analysis revealed that other significant adverse events reported in the male subgroup included rash, illness, dizziness, dialysis, and vomiting. In the female subgroup, nausea was the most common event, followed by diarrhea, fatigue, headache, and alopecia. The female subgroup also reported significant occurrences of abdominal discomfort, illness, dialysis, therapy interrupted, and liver disorder. Detailed results can be found in Figure 3.

**Figure 4.**
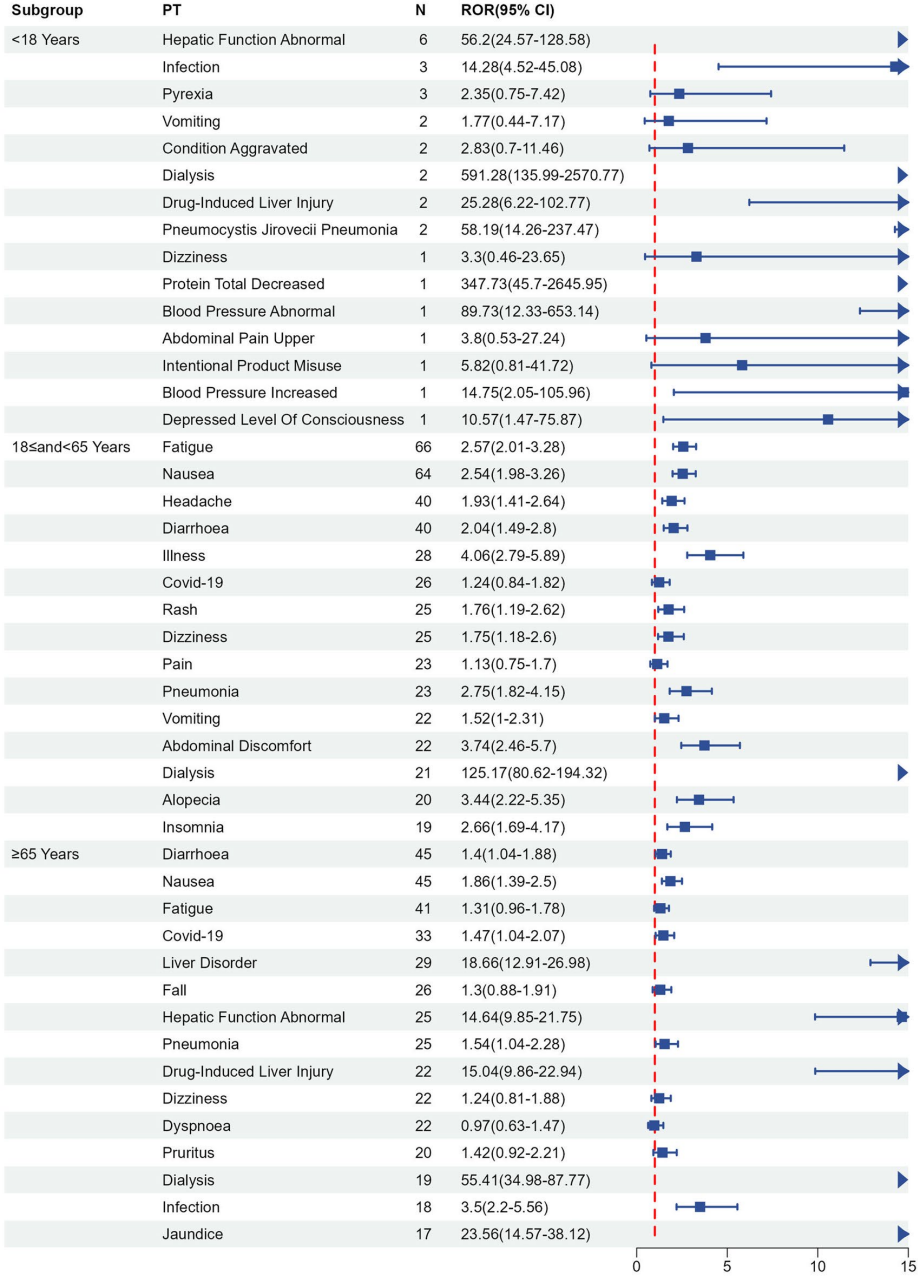
Age-based subgroup analysis of avacopan-related AEs. *Note: N* < 10, to be interpreted with caution.

In the age subgroups, among those under 18 years old, hepatic function abnormal was the most common event, followed by infection and pyrexia. This study included fewer adverse reactions in patients under 18 years old, which may be related to the age characteristics of AAV. In the 18 to 65 years age subgroup, fatigue was the most common event, followed by nausea, headache, diarrhea, and illness. This subgroup also reported significant occurrences of rash, dizziness, pneumonia, vomiting, and abdominal discomfort. In the ≥65 years age subgroup, diarrhea was the most common event, followed by nausea, fatigue, COVID-19, and liver disorder. Further analysis indicated that the ≥65 years age subgroup also reported significant occurrences of hepatic function abnormal, pneumonia, drug-induced liver injury, dialysis, and infection. These subgroup analyses were able to identify similarities and differences between the groups. Moreover, this information is potentially crucial for more detailed clinical management, guiding clinicians to adjust treatment based on the characteristics of specific subgroups. Detailed results can be found in Figure 4.

### 3.4. Clinical prioritization of relevant disproportionality signals

We assessed the clinical priority of 183 AEs, finding that 62 AEs had a moderate clinical priority (33.8%), while 121 AEs had a low clinical priority (66.1%). Table 4 displays those with moderate clinical priority. Among these, the AEs rated 5 points included multiple organ dysfunction syndrome and pulmonary alveolar hemorrhage, while drug-induced liver injury, renal failure, deafness, and fulminant hepatitis were classified as moderate clinical priority DMEs. Detailed results can be found in Table 4.

**Table 4.** AEs ranked as moderate clinical priorities and relevant scores at the PT level.

PT	*N*	ROR (95%Cl)	PRR (χ²)	EBG M (EBGM05)	IC (IC025)	Reporting rate	Case fatality rate	Clinical relevance	Total score	Clinical priority
Multiple Organ Dysfunction Syndrome	9	2.14 (1.11–4.12)	2.14 (5.45)	2.14 (1.24)	1.1 (0.18)	0.13%	88.89%	IME	5	moderate priority
Pulmonary Alveolar Hemorrhage	4	5.75 (2.16–15.36)	5.75 (15.66)	5.74 (2.52)	2.52 (1.23)	0.06%	75.00%	IME	5	moderate priority
Pneumonia	83	2.35 (1.89–2.92)	2.34 (63.61)	2.33 (1.95)	1.22 (0.91)	1.17%	16.87%	IME	4	moderate priority
Drug-Induced Liver Injury	42	9.05 (6.68–12.26)	9 (297.62)	8.97 (6.95)	3.16 (2.72)	0.59%	9.52%	DME	4	moderate priority
Sepsis	26	2.36 (1.61–3.47)	2.36 (20.29)	2.35 (1.71)	1.24 (0.68)	0.36%	34.62%	IME	4	moderate priority
Renal Failure	25	2.13 (1.44–3.15)	2.13 (14.91)	2.12 (1.53)	1.09 (0.52)	0.35%	16.00%	DME	4	moderate priority
*Pneumocystis Jirovecii* Pneumonia	17	11.43 (7.09–18.42)	11.41 (160.55)	11.35 (7.61)	3.5 (2.82)	0.24%	41.18%	IME	4	moderate priority
Interstitial Lung Disease	16	2.89 (1.77–4.71)	2.88 (19.64)	2.88 (1.91)	1.53 (0.82)	0.22%	37.50%	IME	4	moderate priority
Vanishing Bile Duct Syndrome	14	99.88 (58.4–170.84)	99.69 (1,305.25)	95.17 (60.74)	6.57 (5.81)	0.20%	42.86%	IME	4	moderate priority
Deafness	10	3.52 (1.89–6.55)	3.52 (18.01)	3.52 (2.09)	1.81 (0.94)	0.14%	20.00%	DME	4	moderate priority
Bacteraemia	9	6.2 (3.22–11.93)	6.19 (39.06)	6.17 (3.57)	2.63 (1.71)	0.13%	44.44%	IME	4	moderate priority
Device Related Infection	7	5.56 (2.65–11.67)	5.55 (26.07)	5.54 (2.98)	2.47 (1.45)	0.10%	57.14%	None	4	moderate priority
Cytomegalovirus Viraemia	6	8.98 (4.03–20.03)	8.98 (42.34)	8.94 (4.57)	3.16 (2.07)	0.08%	33.33%	IME	4	moderate priority
Drooling	3	4.35 (1.4–13.5)	4.35 (7.72)	4.34 (1.68)	2.12 (0.67)	0.04%	66.67%	None	4	moderate priority
Cholangitis	3	4.35 (1.4–13.52)	4.35 (7.73)	4.35 (1.68)	2.12 (0.67)	0.04%	33.33%	IME	4	moderate priority
Esophageal Candidiasis	3	7.15 (2.3–22.21)	7.15 (15.8)	7.12 (2.76)	2.83 (1.39)	0.04%	33.33%	IME	4	moderate priority
Hepatitis Acute	3	5.23 (1.68–16.23)	5.22 (10.22)	5.21 (2.02)	2.38 (0.94)	0.04%	33.33%	IME	4	moderate priority
Diarrhea	180	2.37 (2.04–2.74)	2.33 (138.41)	2.33 (2.06)	1.22 (1)	2.57%	1.67%	None	3	moderate priority
Nausea	177	2.23 (1.93–2.59)	2.2 (117.68)	2.2 (1.94)	1.14 (0.92)	2.52%	1.13%	None	3	moderate priority
Headache	130	2.04 (1.72–2.43)	2.02 (67.82)	2.02 (1.75)	1.02 (0.76)	1.84%	0.77%	None	3	moderate priority
Abdominal Discomfort	74	3.61 (2.87–4.54)	3.58 (137.76)	3.58 (2.95)	1.84 (1.5)	1.04%	0	None	3	moderate priority
Renal Impairment	38	3.71 (2.69–5.1)	3.69 (74.61)	3.69 (2.82)	1.88 (1.42)	0.53%	5.26%	IME	3	moderate priority
Lower Respiratory Tract Infection	20	3.36 (2.17–5.22)	3.36 (33.07)	3.35 (2.32)	1.75 (1.11)	0.28%	10.00%	IME	3	moderate priority
Thrombosis	18	2.26 (1.42–3.59)	2.25 (12.56)	2.25 (1.53)	1.17 (0.51)	0.25%	0	IME	3	moderate priority
Cholestasis	16	8.11 (4.96–13.26)	8.09 (99.13)	8.07 (5.35)	3.01 (2.31)	0.22%	6.25%	IME	3	moderate priority
Leukopenia	14	2.62 (1.55–4.43)	2.62 (13.98)	2.61 (1.69)	1.39 (0.64)	0.20%	0	IME	3	moderate priority
Deep Vein Thrombosis	14	3.62 (2.14–6.13)	3.62 (26.51)	3.61 (2.33)	1.85 (1.11)	0.20%	14.29%	IME	3	moderate priority
Cytomegalovirus Infection	12	6.29 (3.57–11.09)	6.28 (53.16)	6.27 (3.9)	2.65 (1.85)	0.17%	41.67%	None	3	moderate priority
Covid-19 Pneumonia	9	2.5 (1.3–4.81)	2.5 (8.08)	2.5 (1.44)	1.32 (0.41)	0.13%	22.22%	IME	3	moderate priority
Pulmonary Hemorrhage	9	14.92 (7.74–28.75)	14.9 (115.88)	14.8 (8.55)	3.89 (2.97)	0.13%	11.11%	IME	3	moderate priority
Pneumonia Bacterial	9	6.03 (3.13–11.61)	6.03 (37.63)	6.01 (3.48)	2.59 (1.67)	0.13%	0	IME	3	moderate priority
Hypertransaminasaemia	8	5.65 (2.82–11.32)	5.65 (30.51)	5.63 (3.15)	2.49 (1.53)	0.11%	25.00%	None	3	moderate priority
Hepatotoxicity	7	2.42 (1.15–5.08)	2.42 (5.82)	2.42 (1.3)	1.27 (0.25)	0.10%	0	IME	3	moderate priority
Ageusia	7	2.64 (1.26–5.54)	2.64 (7.12)	2.64 (1.42)	1.4 (0.38)	0.10%	28.57%	None	3	moderate priority
Blood Bilirubin Increased	7	3.24 (1.54–6.8)	3.24 (10.8)	3.23 (1.74)	1.69 (0.67)	0.10%	28.57%	None	3	moderate priority
Haematemesis	7	3.37 (1.61–7.08)	3.37 (11.66)	3.37 (1.81)	1.75 (0.73)	0.10%	14.29%	IME	3	moderate priority
End Stage Renal Disease	7	4.16 (1.98–8.74)	4.16 (16.78)	4.15 (2.23)	2.05 (1.03)	0.10%	14.29%	IME	3	moderate priority
Hyperkalaemia	7	2.18 (1.04–4.57)	2.18 (4.45)	2.18 (1.17)	1.12 (0.1)	0.10%	0	IME	3	moderate priority
Toothache	6	3.28 (1.47–7.31)	3.28 (9.5)	3.28 (1.68)	1.71 (0.62)	0.08%	33.33%	None	3	moderate priority
Head Discomfort	6	2.65 (1.19–5.91)	2.65 (6.17)	2.65 (1.36)	1.41 (0.31)	0.08%	33.33%	None	3	moderate priority
Mouth Swelling	6	8.53 (3.82–19.02)	8.52 (39.67)	8.49 (4.34)	3.09 (1.99)	0.08%	33.33%	None	3	moderate priority
Cerebral Infarction	6	2.94 (1.32–6.55)	2.94 (7.67)	2.94 (1.5)	1.55 (0.46)	0.08%	0	IME	3	moderate priority
Steroid Diabetes	6	23.56 (10.53–52.69)	23.54 (128.04)	23.29 (11.87)	4.54 (3.44)	0.08%	16.67%	IME	3	moderate priority
Gingival Pain	5	6.77 (2.81–16.29)	6.76 (24.48)	6.75 (3.23)	2.75 (1.57)	0.07%	40.00%	None	3	moderate priority
Immunosuppression	5	4.25 (1.77–10.21)	4.24 (12.37)	4.24 (2.03)	2.08 (0.9)	0.07%	20.00%	IME	3	moderate priority
Suspected Drug-Induced Liver Injury	5	110.67 (45 − 272.17)	110.6 (515.61)	105.06 (49.48)	6.72 (5.5)	0.07%	0	IME	3	moderate priority
Hepatitis Cholestatic	4	7.99 (2.99–21.32)	7.98 (24.34)	7.96 (3.5)	2.99 (1.7)	0.06%	0	IME	3	moderate priority
Hypoaesthesia Oral	4	2.79 (1.04–7.43)	2.78 (4.57)	2.78 (1.22)	1.48 (0.18)	0.06%	50.00%	None	3	moderate priority
Urosepsis	4	4 (1.5–10.68)	4 (8.99)	3.99 (1.76)	2 (0.71)	0.06%	0	IME	3	moderate priority
*Clostridium Difficile* Colitis	4	3.64 (1.36–9.71)	3.64 (7.64)	3.63 (1.6)	1.86 (0.57)	0.06%	0	IME	3	moderate priority
Cytomegalovirus Infection Reactivation	4	3.89 (1.46–10.39)	3.89 (8.58)	3.89 (1.71)	1.96 (0.67)	0.06%	0	IME	3	moderate priority
Acute Coronary Syndrome	3	4.97 (1.6–15.43)	4.97 (9.48)	4.96 (1.92)	2.31 (0.86)	0.04%	0	IME	3	moderate priority
Enterococcal Infection	3	5.27 (1.7–16.35)	5.26 (10.34)	5.25 (2.04)	2.39 (0.95)	0.04%	0	IME	3	moderate priority
Escherichia Infection	3	3.13 (1.01–9.71)	3.13 (4.33)	3.12 (1.21)	1.64 (0.2)	0.04%	33.33%	None	3	moderate priority
Pseudomonas Infection	3	2.94 (0.95–9.14)	2.94 (3.85)	2.94 (1.14)	1.56 (0.11)	0.04%	66.67%	IME	3	moderate priority
Near Death Experience	3	4.07 (1.31–12.65)	4.07 (6.94)	4.07 (1.58)	2.02 (0.58)	0.04%	0	IME	3	moderate priority
Chronic Active Epstein-Barr Virus Infection	3	222.87 (67.74–733.26)	222.78 (598.25)	201.31 (74.32)	7.65 (6.12)	0.04%	0	IME	3	moderate priority
Pyelonephritis	3	3.23 (1.04–10.01)	3.23 (4.6)	3.22 (1.25)	1.69 (0.24)	0.04%	0	IME	3	moderate priority
Pneumonia Pneumococcal	3	19.03 (6.1–59.31)	19.02 (50.75)	18.85 (7.28)	4.24 (2.79)	0.04%	0	IME	3	moderate priority
Rectal Ulcer	2	16.91 (4.2–68.01)	16.9 (29.69)	16.78 (5.24)	4.07 (2.39)	0.03%	100.00%	IME	3	moderate priority
Hepatitis Fulminant	2	8.4 (2.1–33.7)	8.4 (12.99)	8.37 (2.62)	3.07 (1.39)	0.03%	50.00%	DME	3	moderate priority
Aortic Dissection	2	7.6 (1.9–30.49)	7.6 (11.43)	7.58 (2.37)	2.92 (1.25)	0.03%	100.00%	IME	3	moderate priority

*Note:* DME, designated medical event; IME, important medical event; *N* < 10, to be interpreted with caution.

### 3.5. Cumulative incidence and time‐to‐onset

**Figure 5.**
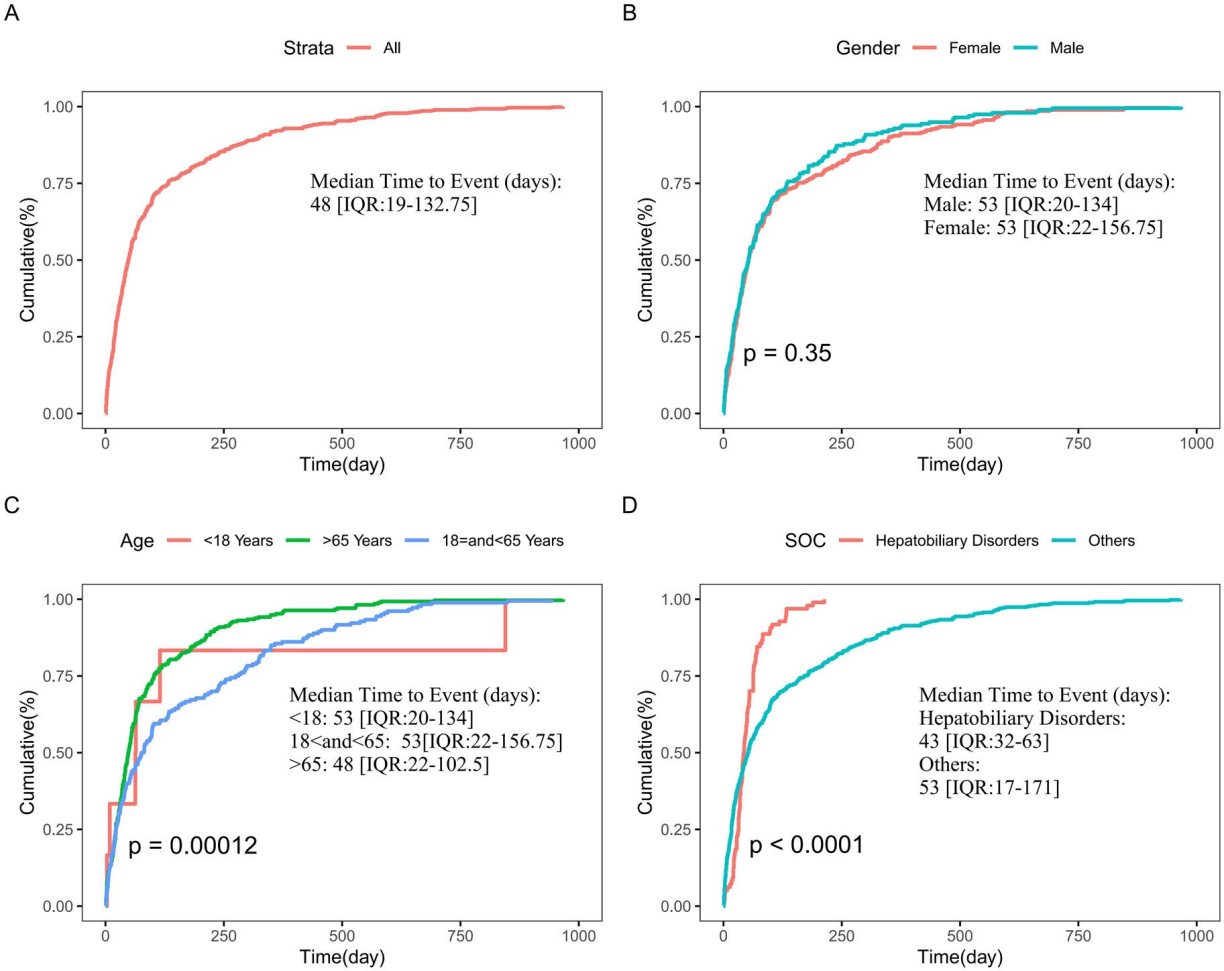
Age/gender/SOC-stratified time-to-onset analysis of avacopan-related AEs. *Note:* IQR, interquartile range.

Among 3,150 reports, 562 included onset-time data. Of these, 197 were male, 274 were female, with only 6 reports in the <18 age group, 180 in the 18–65 age group, and 262 in the >65 age group. There were 82 reports with onset-time data for SOCs classified as hepatobiliary disorders, while other SOCs accounted for 375 reports. Because onset-time data were missing for 82% of reports, the TTO analysis is exploratory and its representativeness is limited; results should be interpreted cautiously. The specific Kaplan-Meier curves are shown in Figure 5. Patients receiving avacopan treatment exhibited significant differences in cumulative incidence across different age groups (log-rank test, *p* < 0.00012), and those who developed hepatobiliary disorders also showed significant differences in cumulative incidence of AEs compared to other SOC levels (log-rank test, *p* < 0.0001). Sex did not have a significant impact on the cumulative incidence of AEs (log-rank test, *p* = 0.35).

### 3.6. Factors affecting hepatobiliary disorders-related AEs of avacopan

We analyzed the correlation between patient sex, age, and concomitant medications with the occurrence of hepatobiliary disorders-related AEs associated with avacopan, as shown in Table 5. Univariate and multivariate logistic regression analyses indicated that age and concomitant medications may be risk factors for hepatobiliary disorders-related AEs associated with avacopan (*p* < 0.05). The multivariate analysis revealed that the risk of hepatobiliary disorders-related AEs increased by 2.89 times when avacopan was administered in combination with rituximab [OR = 2.89 (1.88–4.45), *p* < 0.001], and the risk increased by 4.48 times when administered with proton-pump inhibitors (PPIs) [OR = 4.48 (2.72–7.39), *p* < 0.001]. Compared to the <45 age group, patients aged 65–75 had a 2.71 times increased risk of hepatobiliary disorders-related AEs [OR = 2.71 (1.37–5.36), *p* = 0.004], and patients over 75 years old had a 3.25 times increased risk [OR = 3.25 (1.67–6.32), *p* < 0.001]. No association was found between the concomitant use of cyclophosphamide (CTX) and statins and the occurrence of hepatobiliary disorders-related AEs associated with avacopan (*p* > 0.05).

**Table 5.** Univariate and multivariate logistic regression analysis of the odds ratio for hepatobiliary disorders-related AEs of avacopan.

Variables	Factor	Non-case (*N* = 1,658)	Case (*N* = 143)	OR (univariable)	OR (multivariable)
Age	<45 (reference)	296 (17.9%)	12 (8.4%)		
	45–65	604 (36.4%)	19 (13.3%)	0.78 (0.37–1.62, *p* = 0.499)	0.91 (0.43–1.93, *p* = 0.802)
	65–75	375 (22.6%)	44 (30.8%)	2.89 (1.50–5.58, *p* = 0.002)	2.71 (1.37–5.36, *p* = 0.004)
	>75	383 (23.1%)	68 (47.6%)	4.38 (2.33–8.24, *p* < 0.001)	3.25 (1.67–6.32, *p* < 0.001)
Sex	Female (reference)	964 (58.1%)	99 (69.2%)		
	Male	694 (41.9%)	44 (30.8%)	0.62 (0.43–0.89, *p* = 0.010)	0.49 (0.33–0.74, *p* < 0.001)
Concomitant drug	No (reference)	1,494 (90.1%)	90 (62.9%)		
	RTX	164 (9.9%)	53 (37.1%)	5.36 (3.68–7.81, *p* < 0.001)	2.89 (1.88–4.45, *p* < 0.001)
	No (reference)	1,601 (96.6%)	131 (91.6%)		
	CTX	57 (3.4%)	12 (8.4%)	2.57 (1.35–4.92, *p* = 0.004)	1.36 (0.66–2.79, *p* = 0.405)
	No (reference)	1,581 (95.4%)	97 (67.8%)		
	PPIs	77 (4.6%)	46 (32.2%)	9.74 (6.41–14.80, *p* < 0.001)	4.48 (2.72–7.39, *p* < 0.001)
	No (reference)	1,612 (97.2%)	122 (85.3%)		
	Statins	46 (2.8%)	21 (14.7%)	6.03 (3.49–10.43, *p* < 0.001)	1.46 (0.74–2.85, *p* = 0.273)

*Note:* RTX, rituximab; CTX, Cyclophosphamide; PPIs, proton-pump inhibitors.

## 4. Discussion

To date, our study is the first to utilize the FAERS database for a post-marketing pharmacovigilance analysis of avacopan. This analysis aims to identify potential new adverse reactions, examine the incidence of adverse reactions by sex and age, assess the timing of these reactions, evaluate clinical priority, and investigate risk factors associated with avacopan-induced hepatotoxicity. The findings from this study will assist in identifying potential adverse reactions and highlighting clinically relevant toxicities, providing valuable insights for the safe clinical use of the medication. Avacopan has been available for over 3 years and is gradually being incorporated into clinical practice, demonstrating positive efficacy and a favorable safety profile in treating AAV patients. In a recently published randomized, double-blind, controlled phase III clinical trial [14], in patients receiving avacopan in combination with rituximab, the remission rates at week 26 and week 52 were higher compared to the control group receiving rituximab with prednisone taper. Regarding adverse events, the overall incidence of adverse events in the avacopan group was similar to that of the control group, both at 98.1%. However, the incidence of serious adverse events (SAEs) in the avacopan group was 34.6% (37/107), which is lower than the 39.3% (42/107) observed in the control group. Notably, the incidence of serious infections in the avacopan group was 10.3% (11/107), compared to 14.0% (15/107) in the control group. Additionally, the incidence of SAEs related to liver function abnormalities in the avacopan group was 2.8% (3/107), slightly lower than the 3.7% (4/107) reported in the control group. Although avacopan demonstrates better efficacy and safety compared to the traditional prednisone taper regimen, there is currently a lack of more real-world data to evaluate the safety of avacopan. Therefore, it is essential to analyze the potential adverse reactions to alert clinicians about whether the medication may trigger or exacerbate complications related to AAV.

This study observed that the majority of reports were concentrated in the United States, Japan, and Europe, with most studies related to prevalence originating from these countries. In contrast, data from Asia, Africa, and Latin America are limited [15]. Analysis of patient demographic characteristics showed that the incidence rate was higher in females (37.9%) compared to males (26.3%); however, males predominated in most studies [16], this may be related to the fact that women are more susceptible to the effects of medications. In this study, most reports involved elderly individuals aged 65 and older (27.7%), with an average age of 60.8 years, which may correlate with previous epidemiological studies showing a higher prevalence of illness in older adults [17]. Hospitalization was the primary outcome reported by patients (23.3%), with pneumonia being the most common reason for hospitalization (33 occurrences), Mortality outcomes warrant particular attention, with pneumonia (14 occurrences), COVID-19 (13 occurrences), and infection (9 occurrences) being the three most relevant AEs, Subgroup analysis revealed that females appeared to have a higher incidence of gastrointestinal and hepatic AEs, such as nausea, diarrhea, and liver-related disorders, compared to males. Males, on the other hand, had a higher incidence of fatigue, pneumonia, and sepsis. These differences may be attributed to physiological and pharmacokinetic variations between genders. Younger patients (<18 years) are at higher risk for hepatic function abnormalities and infections, while older patients (≥65 years) are more prone to diarrhea, nausea, and hepatic function abnormalities. The 18 to 65 years subgroup shows a broader range of AEs, including fatigue, nausea, and blood pressure abnormalities. The majority of adverse reactions occurred within 2 months (57.1%), with a median onset time of 48 days (interquartile range [IQR] 19–132.75 days), indicating that the timing of adverse reactions should be monitored to facilitate timely adjustments to medication.

The main finding of our study is that a total of 183 AEs in the FAERS database were identified as meeting at least one signal detection algorithm. Among these, 75 AEs met the criteria for four different signal detection algorithms, We conducted a clinical priority assessment of the 183 AEs, resulting in 62 AEs classified as having moderate priority, which included 4 DMEs and 59 IMEs.

In the clinical priority assessment, multiple organ dysfunction syndrome and pulmonary alveolar hemorrhage received the highest scores, with reported mortality rates of 89% and 75%, respectively. Both conditions are closely associated with complications related to AAV, which is a destructive small-vessel vasculitis that can lead to multi-organ inflammation, including renal involvement [18]. During the course of AAV, multiple organ dysfunction syndrome may develop, further complicating treatment and presenting challenges. The occurrence of MODS may be related to the severity of AAV, the response to treatment, and individual patient differences [19]. Pulmonary alveolar hemorrhage secondary to AAV is a rare and life-threatening condition, characterized by severe respiratory failure [20], it is also associated with high morbidity and mortality rates in patients with AAV. The pathogenesis of pulmonary alveolar hemorrhage in AAV is complex, involving immune-mediated damage to pulmonary capillaries, which is often exacerbated by the presence of anti-neutrophil antibodies targeting neutrophil components [21].

Drug-induced liver injury (DILI), renal failure, deafness, and fulminant hepatitis are considered DMEs with moderate clinical priority. Renal failure is a significant complication of AAV that can lead to end-stage renal disease (ESRD) and severely impact patient survival. Renal involvement in AAV is associated with poor prognosis, and understanding the clinical factors that lead to renal failure is crucial for improving patient management and outcomes [22]. Deafness is an unexpected finding, although no direct relationship between avacopan and deafness has been identified so far, conditions associated with AAV, such as otitis media, may lead to significant hearing loss [23], focused monitoring should be conducted in clinical practice. Hepatobiliary toxicity, including DILI, fulminant hepatitis, and vanishing bile duct syndrome (VBDS), has been a longstanding safety concern regarding avacopan. In the ADVOCATE trial [24], in the avacopan group, 9 out of 166 patients (5.4%) experienced SAE related to abnormal liver function tests. Although most patients had a recovery of liver function after discontinuation of the drug, there are some case reports indicating that the damage may persist long-term. One case involved an elderly female patient with microscopic polyangiitis who developed cholestatic liver injury after 2 months of avacopan therapy, leading to the discontinuation of the drug. Despite stopping avacopan and receiving treatment, her liver enzyme levels remained elevated, and histological examination following a liver biopsy revealed residual inflammation around the portal vein and focal necrosis, indicating that liver injury was still ongoing despite the withdrawal of avacopan [25]. A 10-year-old boy with granulomatosis and polyangiitis presented with diffuse pulmonary hemorrhage. Following a course of methylprednisolone pulse therapy, he received rituximab twice daily and 30 mg of avacopan as primary treatment. On the 31st day of avacopan treatment, abnormal liver function tests were suddenly observed, and liver function improved after discontinuation of the medication [26]. This suggests that avacopan-induced liver injury may occur rapidly in children, necessitating careful monitoring. Another retrospective study evaluated DILI in 22 AAV patients treated with avacopan [27], among them, 9 patients exhibited DILI, with 8 patients showing improvement after discontinuation of the drug, while another patient developed VBDS, which led to death. The causes of VBDS included DILI, and Yamaguchi et al. previously reported cases of VBDS occurring during avacopan treatment [28], the patient was treated with a combination of multiple medications (rituximab, avacopan, prednisolone, and sulfamethoxazole/trimethoprim), and it could not be determined whether avacopan was the sole causative agent of VBDS, the use of multiple drugs complicates the assessment of the causal relationship between a drug and AEs. However, Kojima et al. later reported a case involving a patient with GPA [29], the patient presented with severe liver abnormalities and significant jaundice, and their condition improved after discontinuing avacopan, which did not contain corticosteroids. There was suspicion that the issues were related to avacopan and/or rituximab. This study evaluated 35 AEs under the category of hepatobiliary disorders as outcomes, logistic regression was performed to analyze the risk of avacopan-induced hepatobiliary toxicity based on gender, age, and concomitant drug exposure. The results indicated that both older age and female gender were risk factors for hepatobiliary toxicity, and among concomitant medications, rituximab was found to increase the risk of hepatobiliary toxicity, supporting the hypothesis put forth by Kojima et al. Similarly, Kentaro et al. suggested that older individuals are more susceptible to avacopan-induced liver injury.

Among 183 AEs who met at least one algorithm, infections and infestations were the most frequently reported SOC (38/183), including viral, fungal, and bacterial infections. COVID-19 was the most commonly reported AE. A retrospective study analyzed the outcomes of 8 patients who contracted COVID-19 while receiving avacopan [30], the results indicated that only one patient was hospitalized due to AAV-related interstitial lung disease, and died from respiratory failure secondary to severe COVID-19, while the remaining patients recovered without experiencing long COVID. The complement system plays a crucial role in bacterial and fungal infections, with complement activation ultimately resulting in the cleavage of C5 into C5a and C5b. C5a acts as a potent chemotactic factor, while C5b is a key component in the formation of the membrane attack complex, aiding in bacterial lysis [31]. Therefore, genetic defects in C5 are associated with severe, life-threatening infections caused by encapsulated bacteria, such as *Neisseria meningitidis* and *Streptococcus pneumoniae* [32]. C5a-C5aR1-dependent activation of phagocytes is crucial for fungal clearance and host survival. Mechanistically, C5aR1 mediates neutrophil uptake of fungi and the survival, accumulation, and killing of macrophages, thereby promoting fungal clearance and host survival. For instance, reduced serum C5a levels or suboptimal complement activation due to C5 single nucleotide polymorphisms are independent risk factors for poor outcomes in patients with candidemia [33], this helps explain the susceptibility of patients treated with avacopan to fungal infections. Clinicians should be aware that serious infections may occur after avacopan treatment for AAV, including *Pneumocystis jirovecii* pneumonia, *Cytomegaloviru*s infection, and *Pseudomonas* infections, with reported mortality rates in these AEs exceeding 40%.

Avacopan, as a moderate CYP3A4 inhibitor, was evaluated for drug interactions in a clinical study [34]. In the fasting state, it increased the AUC of the CYP3A4 substrate midazolam by 1.81 times, and simvastatin by 2.6 to 3.5 times, while the AUC of the CYP2C9 substrate celecoxib increased by 1.15 times. Additionally, co-administration with the CYP3A4 inhibitor itraconazole increased the AUC of avacopan by 2.19 times, whereas the CYP3A4 inducer rifampin decreased it by 13.5 times. In this study, univariate logistic regression analysis found that the co-administration of avacopan with statins increased the risk of hepatobiliary toxicity [OR = 6.03 (3.49, 10.43), *p* < 0.001], but this association was not statistically significant after adjusting for other variables [OR = 1.46 (0.74, 2.85), *p* = 0.237], suggesting that other factors may confound this relationship. The severity of underlying diseases, liver function status, or other concomitant medications may play a role in the development of hepatobiliary toxicity. Co-administration of PPIs with avacopan increased the risk of AEs, and this association remained statistically significant after adjusting for other variables [OR = 4.48 (2.72, 7.39), *p* < 0.001]. Although the prescribing information indicates that PPIs, such as omeprazole, are not expected to have a clinically relevant effect on avacopan plasma exposure. Cyclophosphamide is a widely used chemotherapy and immunosuppressive agent that can lead to severe liver damage, with mechanisms of liver toxicity involving oxidative stress, inflammation, and apoptosis. This study found that the combination of avacopan and CTX increased the risk of hepatobiliary toxicity [OR = 2.57 (1.35, 4.92), *p* = 0.004], but this association was not statistically significant after adjusting for other variables [OR = 1.36 (0.66, 2.79), *p* = 0.405], which has been previously discussed regarding its use with RTX. In the context of polypharmacy, which is frequently encountered in patients with ANCA-associated vasculitis due to multimorbidity and complex immunosuppressive regimens, the overlap of toxicity profiles between avacopan and standard AAV treatments warrants closer consideration. Avacopan is often prescribed alongside agents such as corticosteroids, rituximab, CTX, and trimethoprim-sulfamethoxazole, all of which possess their own hepatotoxic or immunosuppressive potential. Although our study could not fully adjust for all interacting medications due to the limitations of the FAERS dataset, existing evidence suggests possible additive or synergistic hepatobiliary risks, particularly with simultaneous use of rituximab. Furthermore, drugs like CTX and high-dose corticosteroids are associated with mitochondrial dysfunction, metabolic liver injury, or reactivation of latent viral infections [35–37], which may exacerbate avacopan-induced hepatotoxicity or infections. Therefore, the cumulative burden of immunosuppressants and hepatotoxic drugs may enhance the susceptibility of certain patients, especially the elderly. Clinical vigilance is warranted, and therapeutic drug monitoring or judicious dose planning may help optimize safety when avacopan is introduced in a polypharmacy setting. Future large-scale clinical studies are needed to clarify whether the co-administration of avacopan with certain drugs increases the risk of hepatobiliary toxicity, to guide clinicians in appropriately adjusting medication regimens.

## 5. Limitations

When conducting a comprehensive analysis of avacopan using the FAERS database, we encountered several limitations. (1) As a spontaneous reporting system, the integrity and accuracy of FAERS data are limited by the voluntary nature of reporting, which may result in underreporting, duplicate reports, and inaccurate information, thereby affecting the reliability of the study results. (2) Due to the lack of information about healthy populations in the FAERS database, we are unable to accurately calculate the incidence of drug-related AEs, nor can we assess potential differences among various populations. (3) The correlation between avacopan and AEs is based on descriptions in the reports rather than a rigorous assessment of causality, and thus we cannot determine whether these signals represent true causal relationships. This study should be considered as a ‘report of correlation strength’ rather than a ‘true risk estimate’ for all signals. The occurrence of AEs may be related to the patient’s underlying diseases, concomitant medications, or other unknown factors. (3) Similarly, the results of TTO require more cautious interpretation due to the large amount of missing data. (4) We are unable to provide definitive evidence to support potential biological mechanisms between avacopan and AEs. Finally, our findings need to be further validated through prospective studies and clinical trials, to confirm the causal relationship between the drug and AEs. This study relied on a spontaneous-reporting database and therefore could not adjust for key potential confounders such as baseline liver function, corticosteroid dosage, or disease activity—factors that are strongly associated with hepatobiliary adverse events and may have led to an overestimation of avacopan-related safety signals. Future investigations should leverage structured electronic health records or MarketScan database that incorporate laboratory values, precise medication dosing, and validated disease-activity scores to enable more robust causal inference. Despite these limitations, the FAERS database remains an important tool for monitoring drug safety and identifying potential signals of adverse drug reactions.

## 6. Conclusions

This study provides a comprehensive insight into the safety of avacopan based on real-world data from the FAERS database. Our analysis identified AEs associated with avacopan, with a particular focus on hepatobiliary disorders, infections, and other clinically significant AEs. The findings underscore the importance of careful monitoring of patients receiving avacopan treatment, especially regarding liver function and potential infections. The results indicate that while avacopan is an effective treatment for AAV, close clinical monitoring should be conducted during its use to manage and mitigate potential adverse events. This study also highlights the need for further research, to better understand the mechanisms underlying these AEs and to identify strategies for their prevention and management.

## Supplementary Material

Revised Supplementary Tables.docx

## Data Availability

Publicly available datasets were analyzed in this study. This data can be found here: All data come from the FAERS database, which is available at https://fis.fda.gov/extensions/FPD-QDE-FAERS/FPD-QDE-FAERS.html.

## References

[1] Jayne DRW, Merkel PA, Schall TJ, et al. Avacopan for the treatment of ANCA-associated vasculitis. N Engl J Med. 2021;384(7):599–609. doi: 10.1056/NEJMoa2023386. 33596356

[2] Nguyen ID, Sinnathamby ES, Mason J, et al. Avacopan, a novel competitive C5a receptor antagonist, for severe antineutrophil cytoplasmic autoantibody-associated vasculitis. Clin Drug Investig. 2023;43(8):595–603. doi: 10.1007/s40261-023-01298-z. 37596445

[3] Harigai M, Takada H. Avacopan, a selective C5a receptor antagonist, for anti-neutrophil cytoplasmic antibody-associated vasculitis. Mod Rheumatol. 2022;32(3):475–483. doi: 10.1093/mr/roab104. 34984461

[4] Lee A. Avacopan: first approval. Drugs. 2022;82(1):79–85. doi: 10.1007/s40265-021-01643-6. 34826105

[5] Tagami G, Yamaguchi M, Sugiyama H, et al. Efficacy and safety of avacopan in antineutrophil cytoplasmic autoantibody-associated vasculitis: a retrospective cohort study in Japan. BMC Rheumatol. 2025;9(1):8–8. doi: 10.1186/s41927-025-00456-4. 39844309 PMC11756139

[6] Gastaldon C, Raschi E, Kane JM, et al. Post-marketing safety concerns with esketamine: a disproportionality analysis of spontaneous reports submitted to the FDA adverse event reporting system. Psychother Psychosom. 2021;90(1):41–48. doi: 10.1159/000510703. 32854103

[7] Bate A, Evans SJW. Quantitative signal detection using spontaneous ADR reporting. Pharmacoepidemiol Drug Saf. 2009;18(6):427–436. 19358225 10.1002/pds.1742

[8] van Puijenbroek EP, Bate A, Leufkens HG, et al. A comparison of measures of disproportionality for signal detection in spontaneous reporting systems for adverse drug reactions. Pharmacoepidemiol Drug Saf. 2002;11(1):3–10. doi: 10.1002/pds.668. 11998548

[9] Evans SJ, Waller PC, Davis S. Use of proportional reporting ratios (PRRs) for signal generation from spontaneous adverse drug reaction reports. Pharmacoepidemiol Drug Saf. 2001;10(6):483–486. doi: 10.1002/pds.677. 11828828

[10] Bate A, Lindquist M, Edwards IR, et al. A Bayesian neural network method for adverse drug reaction signal generation. Eur J Clin Pharmacol. 1998;54(4):315–321. doi: 10.1007/s002280050466. 9696956

[11] Dumouchel W. Bayesian data mining in large frequency tables, with an application to the FDA spontaneous reporting system. Am Stat. 1999;53(3):177–190. doi: 10.1080/00031305.1999.10474456.

[12] Cecco S, Puligheddu S, Fusaroli M, et al. Emerging toxicities of antibody-drug conjugates for breast cancer: clinical prioritization of adverse events from the FDA adverse event reporting system. Target Oncol. 2024;19(3):435–445. doi: 10.1007/s11523-024-01058-9. 38696126 PMC11111510

[13] Sartori D, Aronson JK, Erlanson N, et al. A comparison of signals of designated medical events and non-designated medical events: results from a scoping review. Drug Saf. 2024;47(5):475–485. doi: 10.1007/s40264-024-01403-x. 38401041 PMC11018663

[14] Geetha D, Dua A, Yue H, et al. Efficacy and safety of avacopan in patients with ANCA-associated vasculitis receiving rituximab in a randomised trial. Ann Rheum Dis. 2024;83(2):223–232. doi: 10.1136/ard-2023-224816. 37979959 PMC10850685

[15] Sandino J, Morales E. Role of avacopan on antineutrophil cytoplasmic antibody (ANCA)-associated vasculitis. J Clin Med. 2024;13(22):6676–6676. doi: 10.3390/jcm13226676. 39597819 PMC11594377

[16] Mohammad AJ. An update on the epidemiology of ANCA-associated vasculitis. Rheumatology (Oxford). 2020;59(S3):iii42–iii50. 32348522 10.1093/rheumatology/keaa089

[17] Watts RA, Hatemi G, Burns JC, et al. Global epidemiology of vasculitis. Nat Rev Rheumatol. 2022;18(1):22–34. doi: 10.1038/s41584-021-00718-8. 34853411 PMC8633913

[18] Hakroush S, Kluge IA, Baier E, et al. Relevance of complement C4 deposits localized to distinct vascular compartments in ANCA-associated renal vasculitis. Int J Mol Sci. 2022;23(22):14325. doi: 10.3390/ijms232214325. 36430804 PMC9695342

[19] Molnár A, Studinger P, Ledó N. Diagnostic and therapeutic approach in ANCA-associated glomerulonephritis: a review on management strategies. Front Med (Lausanne). 2022;9:884188. doi: 10.3389/fmed.2022.884188. 35721093 PMC9205443

[20] Delvino P, Monti S, Balduzzi S, et al. The role of extra-corporeal membrane oxygenation (ECMO) in the treatment of diffuse alveolar haemorrhage secondary to ANCA-associated vasculitis: report of two cases and review of the literature. Rheumatol Int. 2019;39(2):367–375. doi: 10.1007/s00296-018-4116-z. 30074077

[21] Cartin-Ceba R, Diaz-Caballero L, Al-Qadi MO, et al. Diffuse alveolar hemorrhage secondary to antineutrophil cytoplasmic antibody-associated vasculitis: predictors of respiratory failure and clinical outcomes. Arthritis Rheumatol. 2016;68(6):1467–1476. doi: 10.1002/art.39562. 26713723

[22] Kronbichler A, Shin JI, Lee KH, et al. Clinical associations of renal involvement in ANCA-associated vasculitis. Autoimmun Rev. 2020;19(4):102495. doi: 10.1016/j.autrev.2020.102495. 32068190

[23] Watanabe T, Yoshida H, Kishibe K, et al. Cochlear implantation in patients with bilateral deafness caused by otitis media with ANCA-associated vasculitis (OMAAV): a report of four cases. Auris Nasus Larynx. 2018;45(5):922–928. doi: 10.1016/j.anl.2017.12.007. 29807863

[24] Jayne DRW, Merkel PA, Schall TJ, et al. Avacopan for the treatment of ANCA-associated vasculitis. N Engl J Med. 2021;384(7):599–609. doi: 10.1056/NEJMoa2023386. 33596356

[25] Yamaguchi H, Fujii N, Shimizu H. Persistent liver injury following avacopan discontinuation in antineutrophil cytoplasmic antibody-associated vasculitis: A rare case of liver biopsy in the chronic phase of liver injury. Mod Rheumatol Case Rep. 2025;9(1):163–167. doi: 10.1093/mrcr/rxae071. 39548758

[26] Nishino T, Tomori S, Haruyama M, et al. A case of rapid avacopan-induced liver injury in pediatric granulomatosis with polyangiitis. Pediatr Nephrol. 2024;39(10):2919–2922. doi: 10.1007/s00467-024-06376-8. 38619581

[27] Mori K, Shirai T, Mutoh T, et al. Drug-induced liver injury related to avacopan therapy. Rheumatology. 2025;64(5):2533–2540. doi: 10.1093/rheumatology/keae689. 39672792

[28] Satoshi Y, Miho Y, Toshiki K, et al. A case of vanishing bile duct syndrome during treatment of microscopic polyangiitis with avacopan. Rheumatology (Oxford). 2023;63(4):e120–e122. doi: 10.1093/rheumatology/kead285. 37307092

[29] Kanako K, Shoichi F, Mai T, et al. Severe prolonged liver abnormality with jaundice during treatment for granulomatosis with polyangiitis with rituximab and avacopan. Rheumatology (Oxford). 2023;63(3):e101–e103. doi: 10.1093/rheumatology/kead509. 37740250

[30] Aqeel F, Zonozi R, Jeyabalan A, et al. Outcomes of COVID-19 in patients with ANCA-associated vasculitis receiving avacopan. RMD Open. 2023;9(3):e003435. doi: 10.1136/rmdopen-2023-003435. 37640515 PMC10462954

[31] Hess C, Kemper C. Complement-mediated regulation of metabolism and basic cellular processes. Immunity. 2016;45(2):240–254. doi: 10.1016/j.immuni.2016.08.003. 27533012 PMC5019180

[32] Rosenfeld SI, Kelly ME, Leddy JP. Hereditary deficiency of the fifth component of complement in man. I. Clinical, immunochemical, and family studies. J Clin Invest. 1976;57(6):1626–1634. doi: 10.1172/JCI108433. 932197 PMC436822

[33] Desai JV, Kumar D, Freiwald T, et al. C5a-licensed phagocytes drive sterilizing immunity during systemic fungal infection. Cell. 2023;186(13):2802–2822.e22. doi: 10.1016/j.cell.2023.04.031. 37220746 PMC10330337

[34] Miao S, Bekker P, Armas D, et al. Pharmacokinetic evaluation of the CYP3A4 and CYP2C9 drug-drug interaction of avacopan in 2 open-label studies in healthy participants. Clin Pharmacol Drug Dev. 2024;13(5):517–533. doi: 10.1002/cpdd.1389. 38423992

[35] Stoffel TJR, Segatto AL, Silva MM, et al. Cyclophosphamide in Drosophila promotes genes and transposable elements differential expression and mitochondrial dysfunction. Comp Biochem Physiol C Toxicol Pharmacol. 2020;230:108718. doi: 10.1016/j.cbpc.2020.108718. 31982542

[36] Niu F, Zhang B, Feng J, et al. Protein profiling identified mitochondrial dysfunction and synaptic abnormalities after dexamethasone intervention in rats with traumatic brain injury. Neural Regen Res. 2021;16(12):2438–2445. doi: 10.4103/1673-5374.313047. 33907032 PMC8374556

[37] Kim CW, Choi KC. Effects of anticancer drugs on the cardiac mitochondrial toxicity and their underlying mechanisms for novel cardiac protective strategies. Life Sci. 2021;277:119607. doi: 10.1016/j.lfs.2021.119607. 33992675

