# Supplementary material for: Post-marketing safety profile and clinical prioritization of adverse events with avacopan in ANCA-associated vasculitis: a FAERS pharmacovigilance analysis
Source: Ren Fail. 2025 Oct 23;47(1):2568971. doi: 10.1080/0886022X.2025.2568971 (PMC12557820; doi:10.1080/0886022X.2025.2568971)
Supplement: Revised Supplementary Tables.docx [file IRNF_A_2568971_SM1622.docx]

**Table S1.** Four major algorithms used for signal detection.

| **Algorithms** | **Equation** | **Criteria** |
| --- | --- | --- |
| ROR | ROR=ad/b/c | lower limit of 95% CI>1, a≥3 |
|  | 95%CI=e^ln(ROR)±1.96(1/a+1/b+1/c+1/d)^0.5^ |  |
| PRR | PRR=a(c+d)/c/(a+b) | PRR≥2, χ^2^≥4, a≥3 |
|  | χ^2^=[(ad-bc)^2](a+b+c+d)/[(a+b)(c+d)(a+c)(b+d)] |  |
| BCPNN | IC=log_2_a(a+b+c+d)(a+c)(a+b) | IC025>0 |
|  | 95%CI= E(IC) ± 2V(IC)^0.5 |  |
| MGPS | EBGM=a(a+b+c+d)/(a+c)/(a+b) | EBGM05>2 |
|  | 95%CI=e^ln(EBGM)±1.96(1/a+1/b+1/c+1/d)^0.5^ |  |

Caption: a, number of reports containing both the target drug and target adverse drug reaction; b, number of reports containing other adverse drug reaction of the target drug; c, number of reports containing the target adverse drug reaction of other drugs; d, number of reports containing other drugs and other adverse drug reactions. 95%CI, 95% confidence interval; χ^2^, chi-squared; IC, information component; IC025, the lower limit of 95% CI of the IC; E(IC), the IC expectations; V(IC), the variance of IC; EBGM, empirical Bayesian geometric mean; EBGM05, the lower limit of 95% CI of EBGM.

**Table S2.** Criteria and relevant scores to prioritize AEs emerged from disproportionality analysis.

| **Criterium** | **2 points** | **1 point** | **0 point** |
| --- | --- | --- | --- |
| Reporting rate (cases/non-cases) | > 10% | 1–10% | 0–1% |
| Signal stability (consistency across disproportionality analyses) | 3 of 3 | 2 of 3 | 1 of 3 |
| Reported case fatality rate (proportion of reports with death as outcome) | > 50% | 25–50% | < 25% |
| Clinical relevance (serious likely drug-attributable AEs) | DME | IME | None |

Caption: AEs, adverse events; DME, designated medical event; IME, important medical event.

**Table S3.** AEs associated with death and hospitalization at the PT level.

| **Outcome** | **N** |
| --- | --- |
| **Hospitalization** |  |
| pneumonia | 33 |
| nausea | 25 |
| COVID-19 | 23 |
| dialysis | 20 |
| fall | 20 |
| hepatic function abnormal | 19 |
| therapy interrupted | 19 |
| diarrhoea | 18 |
| fatigue | 18 |
| liver disorder | 18 |
| **Death** |  |
| pneumonia | 14 |
| COVID-19 | 13 |
| infection | 9 |
| sepsis | 9 |
| hepatic function abnormal | 8 |
| multiple organ dysfunction syndrome | 8 |
| dyspnoea | 7 |
| pneumocystis jirovecii pneumonia | 7 |
| cardiac arrest | 6 |
| interstitial lung disease | 6 |

**Table S4.** Clinical prioritization assessment of AEs meeting at least one algorithm at the PT level.

| **PT** | **N** | **ROR(95%Cl)** | **PRR(χ²)** | **EBGM(EBGM05)** | **IC(IC025)** | **Reporting rate** | **Case fatality rate** | **Clinical relevance** | **Total score** | **Clinical priority** |
| --- | --- | --- | --- | --- | --- | --- | --- | --- | --- | --- |
| Multiple Organ Dysfunction Syndrome | 9 | 2.14 ( 1.11 - 4.12 ) | 2.14 ( 5.45 ) | 2.14 ( 1.24 ) | 1.1 ( 0.18 ) | 0.13% | 88.89% | IME | 5 | moderate priority |
| Pulmonary Alveolar Haemorrhage | 4 | 5.75 ( 2.16 - 15.36 ) | 5.75 ( 15.66 ) | 5.74 ( 2.52 ) | 2.52 ( 1.23 ) | 0.06% | 75.00% | IME | 5 | moderate priority |
| Pneumonia | 83 | 2.35 ( 1.89 - 2.92 ) | 2.34 ( 63.61 ) | 2.33 ( 1.95 ) | 1.22 ( 0.91 ) | 1.17% | 16.87% | IME | 4 | moderate priority |
| Drug-Induced Liver Injury | 42 | 9.05 ( 6.68 - 12.26 ) | 9 ( 297.62 ) | 8.97 ( 6.95 ) | 3.16 ( 2.72 ) | 0.59% | 9.52% | DME | 4 | moderate priority |
| Sepsis | 26 | 2.36 ( 1.61 - 3.47 ) | 2.36 ( 20.29 ) | 2.35 ( 1.71 ) | 1.24 ( 0.68 ) | 0.36% | 34.62% | IME | 4 | moderate priority |
| Renal Failure | 25 | 2.13 ( 1.44 - 3.15 ) | 2.13 ( 14.91 ) | 2.12 ( 1.53 ) | 1.09 ( 0.52 ) | 0.35% | 16.00% | DME | 4 | moderate priority |
| Pneumocystis Jirovecii Pneumonia | 17 | 11.43 ( 7.09 - 18.42 ) | 11.41 ( 160.55 ) | 11.35 ( 7.61 ) | 3.5 ( 2.82 ) | 0.24% | 41.18% | IME | 4 | moderate priority |
| Interstitial Lung Disease | 16 | 2.89 ( 1.77 - 4.71 ) | 2.88 ( 19.64 ) | 2.88 ( 1.91 ) | 1.53 ( 0.82 ) | 0.22% | 37.50% | IME | 4 | moderate priority |
| Vanishing Bile Duct Syndrome | 14 | 99.88 ( 58.4 - 170.84 ) | 99.69 ( 1305.25 ) | 95.17 ( 60.74 ) | 6.57 ( 5.81 ) | 0.20% | 42.86% | IME | 4 | moderate priority |
| Deafness | 10 | 3.52 ( 1.89 - 6.55 ) | 3.52 ( 18.01 ) | 3.52 ( 2.09 ) | 1.81 ( 0.94 ) | 0.14% | 20.00% | DME | 4 | moderate priority |
| Bacteraemia | 9 | 6.2 ( 3.22 - 11.93 ) | 6.19 ( 39.06 ) | 6.17 ( 3.57 ) | 2.63 ( 1.71 ) | 0.13% | 44.44% | IME | 4 | moderate priority |
| Device Related Infection | 7 | 5.56 ( 2.65 - 11.67 ) | 5.55 ( 26.07 ) | 5.54 ( 2.98 ) | 2.47 ( 1.45 ) | 0.10% | 57.14% | None | 4 | moderate priority |
| Cytomegalovirus Viraemia | 6 | 8.98 ( 4.03 - 20.03 ) | 8.98 ( 42.34 ) | 8.94 ( 4.57 ) | 3.16 ( 2.07 ) | 0.08% | 33.33% | IME | 4 | moderate priority |
| Drooling | 3 | 4.35 ( 1.4 - 13.5 ) | 4.35 ( 7.72 ) | 4.34 ( 1.68 ) | 2.12 ( 0.67 ) | 0.04% | 66.67% | None | 4 | moderate priority |
| Cholangitis | 3 | 4.35 ( 1.4 - 13.52 ) | 4.35 ( 7.73 ) | 4.35 ( 1.68 ) | 2.12 ( 0.67 ) | 0.04% | 33.33% | IME | 4 | moderate priority |
| Oesophageal Candidiasis | 3 | 7.15 ( 2.3 - 22.21 ) | 7.15 ( 15.8 ) | 7.12 ( 2.76 ) | 2.83 ( 1.39 ) | 0.04% | 33.33% | IME | 4 | moderate priority |
| Hepatitis Acute | 3 | 5.23 ( 1.68 - 16.23 ) | 5.22 ( 10.22 ) | 5.21 ( 2.02 ) | 2.38 ( 0.94 ) | 0.04% | 33.33% | IME | 4 | moderate priority |
| Diarrhoea | 180 | 2.37 ( 2.04 - 2.74 ) | 2.33 ( 138.41 ) | 2.33 ( 2.06 ) | 1.22 ( 1 ) | 2.57% | 1.67% | None | 3 | moderate priority |
| Nausea | 177 | 2.23 ( 1.93 - 2.59 ) | 2.2 ( 117.68 ) | 2.2 ( 1.94 ) | 1.14 ( 0.92 ) | 2.52% | 1.13% | None | 3 | moderate priority |
| Headache | 130 | 2.04 ( 1.72 - 2.43 ) | 2.02 ( 67.82 ) | 2.02 ( 1.75 ) | 1.02 ( 0.76 ) | 1.84% | 0.77% | None | 3 | moderate priority |
| Abdominal Discomfort | 74 | 3.61 ( 2.87 - 4.54 ) | 3.58 ( 137.76 ) | 3.58 ( 2.95 ) | 1.84 ( 1.5 ) | 1.04% | 0 | None | 3 | moderate priority |
| Renal Impairment | 38 | 3.71 ( 2.69 - 5.1 ) | 3.69 ( 74.61 ) | 3.69 ( 2.82 ) | 1.88 ( 1.42 ) | 0.53% | 5.26% | IME | 3 | moderate priority |
| Lower Respiratory Tract Infection | 20 | 3.36 ( 2.17 - 5.22 ) | 3.36 ( 33.07 ) | 3.35 ( 2.32 ) | 1.75 ( 1.11 ) | 0.28% | 10.00% | IME | 3 | moderate priority |
| Thrombosis | 18 | 2.26 ( 1.42 - 3.59 ) | 2.25 ( 12.56 ) | 2.25 ( 1.53 ) | 1.17 ( 0.51 ) | 0.25% | 0 | IME | 3 | moderate priority |
| Cholestasis | 16 | 8.11 ( 4.96 - 13.26 ) | 8.09 ( 99.13 ) | 8.07 ( 5.35 ) | 3.01 ( 2.31 ) | 0.22% | 6.25% | IME | 3 | moderate priority |
| Leukopenia | 14 | 2.62 ( 1.55 - 4.43 ) | 2.62 ( 13.98 ) | 2.61 ( 1.69 ) | 1.39 ( 0.64 ) | 0.20% | 0 | IME | 3 | moderate priority |
| Deep Vein Thrombosis | 14 | 3.62 ( 2.14 - 6.13 ) | 3.62 ( 26.51 ) | 3.61 ( 2.33 ) | 1.85 ( 1.11 ) | 0.20% | 14.29% | IME | 3 | moderate priority |
| Cytomegalovirus Infection | 12 | 6.29 ( 3.57 - 11.09 ) | 6.28 ( 53.16 ) | 6.27 ( 3.9 ) | 2.65 ( 1.85 ) | 0.17% | 41.67% | None | 3 | moderate priority |
| Covid-19 Pneumonia | 9 | 2.5 ( 1.3 - 4.81 ) | 2.5 ( 8.08 ) | 2.5 ( 1.44 ) | 1.32 ( 0.41 ) | 0.13% | 22.22% | IME | 3 | moderate priority |
| Pulmonary Haemorrhage | 9 | 14.92 ( 7.74 - 28.75 ) | 14.9 ( 115.88 ) | 14.8 ( 8.55 ) | 3.89 ( 2.97 ) | 0.13% | 11.11% | IME | 3 | moderate priority |
| Pneumonia Bacterial | 9 | 6.03 ( 3.13 - 11.61 ) | 6.03 ( 37.63 ) | 6.01 ( 3.48 ) | 2.59 ( 1.67 ) | 0.13% | 0 | IME | 3 | moderate priority |
| Hypertransaminasaemia | 8 | 5.65 ( 2.82 - 11.32 ) | 5.65 ( 30.51 ) | 5.63 ( 3.15 ) | 2.49 ( 1.53 ) | 0.11% | 25.00% | None | 3 | moderate priority |
| Hepatotoxicity | 7 | 2.42 ( 1.15 - 5.08 ) | 2.42 ( 5.82 ) | 2.42 ( 1.3 ) | 1.27 ( 0.25 ) | 0.10% | 0 | IME | 3 | moderate priority |
| Ageusia | 7 | 2.64 ( 1.26 - 5.54 ) | 2.64 ( 7.12 ) | 2.64 ( 1.42 ) | 1.4 ( 0.38 ) | 0.10% | 28.57% | None | 3 | moderate priority |
| Blood Bilirubin Increased | 7 | 3.24 ( 1.54 - 6.8 ) | 3.24 ( 10.8 ) | 3.23 ( 1.74 ) | 1.69 ( 0.67 ) | 0.10% | 28.57% | None | 3 | moderate priority |
| Haematemesis | 7 | 3.37 ( 1.61 - 7.08 ) | 3.37 ( 11.66 ) | 3.37 ( 1.81 ) | 1.75 ( 0.73 ) | 0.10% | 14.29% | IME | 3 | moderate priority |
| End Stage Renal Disease | 7 | 4.16 ( 1.98 - 8.74 ) | 4.16 ( 16.78 ) | 4.15 ( 2.23 ) | 2.05 ( 1.03 ) | 0.10% | 14.29% | IME | 3 | moderate priority |
| Hyperkalaemia | 7 | 2.18 ( 1.04 - 4.57 ) | 2.18 ( 4.45 ) | 2.18 ( 1.17 ) | 1.12 ( 0.1 ) | 0.10% | 0 | IME | 3 | moderate priority |
| Toothache | 6 | 3.28 ( 1.47 - 7.31 ) | 3.28 ( 9.5 ) | 3.28 ( 1.68 ) | 1.71 ( 0.62 ) | 0.08% | 33.33% | None | 3 | moderate priority |
| Head Discomfort | 6 | 2.65 ( 1.19 - 5.91 ) | 2.65 ( 6.17 ) | 2.65 ( 1.36 ) | 1.41 ( 0.31 ) | 0.08% | 33.33% | None | 3 | moderate priority |
| Mouth Swelling | 6 | 8.53 ( 3.82 - 19.02 ) | 8.52 ( 39.67 ) | 8.49 ( 4.34 ) | 3.09 ( 1.99 ) | 0.08% | 33.33% | None | 3 | moderate priority |
| Cerebral Infarction | 6 | 2.94 ( 1.32 - 6.55 ) | 2.94 ( 7.67 ) | 2.94 ( 1.5 ) | 1.55 ( 0.46 ) | 0.08% | 0 | IME | 3 | moderate priority |
| Steroid Diabetes | 6 | 23.56 ( 10.53 - 52.69 ) | 23.54 ( 128.04 ) | 23.29 ( 11.87 ) | 4.54 ( 3.44 ) | 0.08% | 16.67% | IME | 3 | moderate priority |
| Gingival Pain | 5 | 6.77 ( 2.81 - 16.29 ) | 6.76 ( 24.48 ) | 6.75 ( 3.23 ) | 2.75 ( 1.57 ) | 0.07% | 40.00% | None | 3 | moderate priority |
| Immunosuppression | 5 | 4.25 ( 1.77 - 10.21 ) | 4.24 ( 12.37 ) | 4.24 ( 2.03 ) | 2.08 ( 0.9 ) | 0.07% | 20.00% | IME | 3 | moderate priority |
| Suspected Drug-Induced Liver Injury | 5 | 110.67 ( 45 - 272.17 ) | 110.6 ( 515.61 ) | 105.06 ( 49.48 ) | 6.72 ( 5.5 ) | 0.07% | 0 | IME | 3 | moderate priority |
| Hepatitis Cholestatic | 4 | 7.99 ( 2.99 - 21.32 ) | 7.98 ( 24.34 ) | 7.96 ( 3.5 ) | 2.99 ( 1.7 ) | 0.06% | 0 | IME | 3 | moderate priority |
| Hypoaesthesia Oral | 4 | 2.79 ( 1.04 - 7.43 ) | 2.78 ( 4.57 ) | 2.78 ( 1.22 ) | 1.48 ( 0.18 ) | 0.06% | 50.00% | None | 3 | moderate priority |
| Urosepsis | 4 | 4 ( 1.5 - 10.68 ) | 4 ( 8.99 ) | 3.99 ( 1.76 ) | 2 ( 0.71 ) | 0.06% | 0 | IME | 3 | moderate priority |
| Clostridium Difficile Colitis | 4 | 3.64 ( 1.36 - 9.71 ) | 3.64 ( 7.64 ) | 3.63 ( 1.6 ) | 1.86 ( 0.57 ) | 0.06% | 0 | IME | 3 | moderate priority |
| Cytomegalovirus Infection Reactivation | 4 | 3.89 ( 1.46 - 10.39 ) | 3.89 ( 8.58 ) | 3.89 ( 1.71 ) | 1.96 ( 0.67 ) | 0.06% | 0 | IME | 3 | moderate priority |
| Acute Coronary Syndrome | 3 | 4.97 ( 1.6 - 15.43 ) | 4.97 ( 9.48 ) | 4.96 ( 1.92 ) | 2.31 ( 0.86 ) | 0.04% | 0 | IME | 3 | moderate priority |
| Enterococcal Infection | 3 | 5.27 ( 1.7 - 16.35 ) | 5.26 ( 10.34 ) | 5.25 ( 2.04 ) | 2.39 ( 0.95 ) | 0.04% | 0 | IME | 3 | moderate priority |
| Escherichia Infection | 3 | 3.13 ( 1.01 - 9.71 ) | 3.13 ( 4.33 ) | 3.12 ( 1.21 ) | 1.64 ( 0.2 ) | 0.04% | 33.33% | None | 3 | moderate priority |
| Pseudomonas Infection | 3 | 2.94 ( 0.95 - 9.14 ) | 2.94 ( 3.85 ) | 2.94 ( 1.14 ) | 1.56 ( 0.11 ) | 0.04% | 66.67% | IME | 3 | moderate priority |
| Near Death Experience | 3 | 4.07 ( 1.31 - 12.65 ) | 4.07 ( 6.94 ) | 4.07 ( 1.58 ) | 2.02 ( 0.58 ) | 0.04% | 0 | IME | 3 | moderate priority |
| Chronic Active Epstein-Barr Virus Infection | 3 | 222.87 ( 67.74 - 733.26 ) | 222.78 ( 598.25 ) | 201.31 ( 74.32 ) | 7.65 ( 6.12 ) | 0.04% | 0 | IME | 3 | moderate priority |
| Pyelonephritis | 3 | 3.23 ( 1.04 - 10.01 ) | 3.23 ( 4.6 ) | 3.22 ( 1.25 ) | 1.69 ( 0.24 ) | 0.04% | 0 | IME | 3 | moderate priority |
| Pneumonia Pneumococcal | 3 | 19.03 ( 6.1 - 59.31 ) | 19.02 ( 50.75 ) | 18.85 ( 7.28 ) | 4.24 ( 2.79 ) | 0.04% | 0 | IME | 3 | moderate priority |
| Rectal Ulcer | 2 | 16.91 ( 4.2 - 68.01 ) | 16.9 ( 29.69 ) | 16.78 ( 5.24 ) | 4.07 ( 2.39 ) | 0.03% | 100.00% | IME | 3 | moderate priority |
| Hepatitis Fulminant | 2 | 8.4 ( 2.1 - 33.7 ) | 8.4 ( 12.99 ) | 8.37 ( 2.62 ) | 3.07 ( 1.39 ) | 0.03% | 50.00% | DME | 3 | moderate priority |
| Aortic Dissection | 2 | 7.6 ( 1.9 - 30.49 ) | 7.6 ( 11.43 ) | 7.58 ( 2.37 ) | 2.92 ( 1.25 ) | 0.03% | 100.00% | IME | 3 | moderate priority |
| Fatigue | 185 | 1.98 ( 1.71 - 2.29 ) | 1.95 ( 87.13 ) | 1.95 ( 1.73 ) | 0.97 ( 0.75 ) | 2.64% | 0.54% | None | 2 | low priority |
| Covid-19 | 117 | 1.84 ( 1.53 - 2.21 ) | 1.83 ( 44.29 ) | 1.83 ( 1.57 ) | 0.87 ( 0.6 ) | 1.65% | 11.11% | None | 2 | low priority |
| Dizziness | 79 | 1.6 ( 1.28 - 2 ) | 1.59 ( 17.55 ) | 1.59 ( 1.32 ) | 0.67 ( 0.35 ) | 1.11% | 1.27% | None | 2 | low priority |
| Rash | 72 | 1.44 ( 1.14 - 1.82 ) | 1.43 ( 9.55 ) | 1.43 ( 1.18 ) | 0.52 ( 0.18 ) | 1.01% | 0 | None | 2 | low priority |
| Illness | 70 | 2.5 ( 1.97 - 3.16 ) | 2.48 ( 62.08 ) | 2.48 ( 2.04 ) | 1.31 ( 0.97 ) | 0.98% | 4.29% | None | 2 | low priority |
| Hepatic Function Abnormal | 63 | 14.53 ( 11.33 - 18.64 ) | 14.41 ( 781.5 ) | 14.32 ( 11.63 ) | 3.84 ( 3.48 ) | 0.88% | 12.70% | None | 2 | low priority |
| Hypertension | 62 | 2.63 ( 2.05 - 3.38 ) | 2.62 ( 62.18 ) | 2.62 ( 2.12 ) | 1.39 ( 1.02 ) | 0.87% | 1.61% | None | 2 | low priority |
| Dialysis | 59 | 61.9 ( 47.73 - 80.27 ) | 61.4 ( 3405.44 ) | 59.67 ( 48 ) | 5.9 ( 5.52 ) | 0.83% | 3.39% | None | 2 | low priority |
| Liver Disorder | 57 | 11.58 ( 8.92 - 15.04 ) | 11.49 ( 543.51 ) | 11.44 ( 9.19 ) | 3.52 ( 3.13 ) | 0.80% | 8.77% | None | 2 | low priority |
| Infection | 55 | 3.01 ( 2.31 - 3.93 ) | 3 ( 73.3 ) | 2.99 ( 2.4 ) | 1.58 ( 1.2 ) | 0.77% | 16.36% | None | 2 | low priority |
| Alopecia | 50 | 2.56 ( 1.94 - 3.38 ) | 2.55 ( 47.07 ) | 2.55 ( 2.02 ) | 1.35 ( 0.94 ) | 0.70% | 2.00% | None | 2 | low priority |
| Abdominal Pain Upper | 48 | 2.23 ( 1.68 - 2.96 ) | 2.22 ( 32.28 ) | 2.22 ( 1.75 ) | 1.15 ( 0.74 ) | 0.67% | 2.08% | None | 2 | low priority |
| Peripheral Swelling | 48 | 2.23 ( 1.68 - 2.96 ) | 2.22 ( 32.18 ) | 2.22 ( 1.75 ) | 1.15 ( 0.73 ) | 0.67% | 0 | None | 2 | low priority |
| Hepatic Enzyme Increased | 47 | 5.48 ( 4.11 - 7.3 ) | 5.45 ( 170.47 ) | 5.44 ( 4.28 ) | 2.44 ( 2.02 ) | 0.66% | 2.13% | None | 2 | low priority |
| Therapy Interrupted | 45 | 2.33 ( 1.74 - 3.13 ) | 2.33 ( 34.06 ) | 2.32 ( 1.82 ) | 1.22 ( 0.79 ) | 0.63% | 2.22% | None | 2 | low priority |
| Neutropenia | 42 | 1.96 ( 1.45 - 2.66 ) | 1.96 ( 19.68 ) | 1.96 ( 1.52 ) | 0.97 ( 0.53 ) | 0.59% | 0 | IME | 2 | low priority |
| Surgery | 38 | 5.36 ( 3.9 - 7.38 ) | 5.34 ( 133.74 ) | 5.33 ( 4.08 ) | 2.41 ( 1.95 ) | 0.53% | 0 | None | 2 | low priority |
| Swelling Face | 35 | 5.97 ( 4.28 - 8.32 ) | 5.94 ( 143.61 ) | 5.93 ( 4.49 ) | 2.57 ( 2.08 ) | 0.49% | 0 | None | 2 | low priority |
| Gastrointestinal Disorder | 31 | 2.51 ( 1.76 - 3.57 ) | 2.5 ( 28.02 ) | 2.5 ( 1.86 ) | 1.32 ( 0.81 ) | 0.43% | 3.23% | None | 2 | low priority |
| Jaundice | 28 | 16.2 ( 11.16 - 23.51 ) | 16.14 ( 394.58 ) | 16.02 ( 11.73 ) | 4 ( 3.46 ) | 0.39% | 21.43% | None | 2 | low priority |
| Renal Disorder | 26 | 5.23 ( 3.56 - 7.7 ) | 5.22 ( 88.5 ) | 5.21 ( 3.77 ) | 2.38 ( 1.82 ) | 0.36% | 0 | None | 2 | low priority |
| Alanine Aminotransferase Increased | 25 | 4.76 ( 3.21 - 7.05 ) | 4.74 ( 73.71 ) | 4.73 ( 3.41 ) | 2.24 ( 1.68 ) | 0.35% | 4.00% | None | 2 | low priority |
| Therapy Cessation | 25 | 4 ( 2.7 - 5.93 ) | 3.99 ( 55.94 ) | 3.98 ( 2.87 ) | 1.99 ( 1.43 ) | 0.35% | 0 | None | 2 | low priority |
| Liver Function Test Increased | 24 | 7.72 ( 5.17 - 11.53 ) | 7.7 ( 139.37 ) | 7.67 ( 5.48 ) | 2.94 ( 2.36 ) | 0.34% | 0 | None | 2 | low priority |
| Blood Creatinine Increased | 23 | 3.53 ( 2.34 - 5.31 ) | 3.52 ( 41.41 ) | 3.51 ( 2.49 ) | 1.81 ( 1.22 ) | 0.32% | 0 | None | 2 | low priority |
| Aspartate Aminotransferase Increased | 22 | 4.91 ( 3.23 - 7.47 ) | 4.9 ( 68.2 ) | 4.89 ( 3.45 ) | 2.29 ( 1.69 ) | 0.31% | 0 | None | 2 | low priority |
| Dysphagia | 22 | 2.28 ( 1.5 - 3.47 ) | 2.28 ( 15.78 ) | 2.28 ( 1.6 ) | 1.19 ( 0.58 ) | 0.31% | 4.55% | None | 2 | low priority |
| Epistaxis | 19 | 2.78 ( 1.77 - 4.35 ) | 2.77 ( 21.49 ) | 2.77 ( 1.9 ) | 1.47 ( 0.82 ) | 0.27% | 0 | None | 2 | low priority |
| Lung Disorder | 18 | 3.21 ( 2.02 - 5.11 ) | 3.21 ( 27.35 ) | 3.21 ( 2.18 ) | 1.68 ( 1.02 ) | 0.25% | 16.67% | None | 2 | low priority |
| Liver Function Test Abnormal | 16 | 10.82 ( 6.61 - 17.69 ) | 10.79 ( 141.49 ) | 10.74 ( 7.12 ) | 3.43 ( 2.72 ) | 0.22% | 0 | None | 2 | low priority |
| Flatulence | 13 | 2.29 ( 1.33 - 3.95 ) | 2.29 ( 9.46 ) | 2.29 ( 1.45 ) | 1.2 ( 0.42 ) | 0.18% | 0 | None | 2 | low priority |
| Blood Pressure Abnormal | 13 | 4.84 ( 2.8 - 8.34 ) | 4.83 ( 39.39 ) | 4.82 ( 3.06 ) | 2.27 ( 1.5 ) | 0.18% | 0 | None | 2 | low priority |
| Fluid Retention | 12 | 2.49 ( 1.41 - 4.38 ) | 2.48 ( 10.62 ) | 2.48 ( 1.54 ) | 1.31 ( 0.51 ) | 0.17% | 8.33% | None | 2 | low priority |
| Fungal Infection | 12 | 3.22 ( 1.83 - 5.67 ) | 3.22 ( 18.3 ) | 3.21 ( 2 ) | 1.68 ( 0.88 ) | 0.17% | 16.67% | None | 2 | low priority |
| C-Reactive Protein Increased | 12 | 2.35 ( 1.33 - 4.14 ) | 2.34 ( 9.25 ) | 2.34 ( 1.46 ) | 1.23 ( 0.43 ) | 0.17% | 0 | None | 2 | low priority |
| Increased Appetite | 12 | 6.11 ( 3.46 - 10.77 ) | 6.1 ( 51 ) | 6.08 ( 3.78 ) | 2.6 ( 1.8 ) | 0.17% | 0 | None | 2 | low priority |
| Respiratory Failure | 11 | 1.8 ( 1 - 3.25 ) | 1.8 ( 3.9 ) | 1.8 ( 1.1 ) | 0.85 ( 0.01 ) | 0.15% | 45.45% | IME | 2 | low priority |
| Laboratory Test Abnormal | 10 | 3.09 ( 1.66 - 5.74 ) | 3.08 ( 14.07 ) | 3.08 ( 1.83 ) | 1.62 ( 0.75 ) | 0.14% | 0 | None | 2 | low priority |
| Glomerular Filtration Rate Decreased | 10 | 5.78 ( 3.11 - 10.76 ) | 5.78 ( 39.39 ) | 5.76 ( 3.43 ) | 2.53 ( 1.65 ) | 0.14% | 0 | None | 2 | low priority |
| Proteinuria | 9 | 3.82 ( 1.98 - 7.34 ) | 3.81 ( 18.65 ) | 3.81 ( 2.2 ) | 1.93 ( 1.01 ) | 0.13% | 11.11% | None | 2 | low priority |
| Hospice Care | 9 | 5.35 ( 2.78 - 10.29 ) | 5.34 ( 31.68 ) | 5.33 ( 3.08 ) | 2.41 ( 1.5 ) | 0.13% | 11.11% | None | 2 | low priority |
| Pollakiuria | 9 | 2.21 ( 1.15 - 4.26 ) | 2.21 ( 5.97 ) | 2.21 ( 1.28 ) | 1.14 ( 0.23 ) | 0.13% | 0 | None | 2 | low priority |
| Localised Infection | 9 | 3.19 ( 1.66 - 6.13 ) | 3.19 ( 13.49 ) | 3.18 ( 1.84 ) | 1.67 ( 0.76 ) | 0.13% | 11.11% | None | 2 | low priority |
| Haematuria | 9 | 3.3 ( 1.72 - 6.36 ) | 3.3 ( 14.42 ) | 3.3 ( 1.91 ) | 1.72 ( 0.81 ) | 0.13% | 11.11% | None | 2 | low priority |
| Sinus Disorder | 8 | 3.5 ( 1.75 - 7 ) | 3.5 ( 14.24 ) | 3.49 ( 1.95 ) | 1.8 ( 0.84 ) | 0.11% | 12.50% | None | 2 | low priority |
| Pharyngeal Swelling | 8 | 3.82 ( 1.91 - 7.66 ) | 3.82 ( 16.64 ) | 3.82 ( 2.14 ) | 1.93 ( 0.97 ) | 0.11% | 0 | None | 2 | low priority |
| Product Use Complaint | 8 | 2.54 ( 1.27 - 5.08 ) | 2.54 ( 7.45 ) | 2.54 ( 1.42 ) | 1.34 ( 0.38 ) | 0.11% | 0 | None | 2 | low priority |
| Rehabilitation Therapy | 8 | 12.19 ( 6.08 - 24.43 ) | 12.18 ( 81.6 ) | 12.11 ( 6.77 ) | 3.6 ( 2.63 ) | 0.11% | 0 | None | 2 | low priority |
| Blood Alkaline Phosphatase Increased | 7 | 4.22 ( 2.01 - 8.86 ) | 4.22 ( 17.14 ) | 4.21 ( 2.26 ) | 2.07 ( 1.05 ) | 0.10% | 0 | None | 2 | low priority |
| Haemoptysis | 7 | 2.88 ( 1.37 - 6.05 ) | 2.88 ( 8.58 ) | 2.88 ( 1.55 ) | 1.52 ( 0.5 ) | 0.10% | 0 | None | 2 | low priority |
| Knee Arthroplasty | 7 | 2.22 ( 1.06 - 4.67 ) | 2.22 ( 4.71 ) | 2.22 ( 1.19 ) | 1.15 ( 0.13 ) | 0.10% | 0 | None | 2 | low priority |
| Nonspecific Reaction | 7 | 7.67 ( 3.65 - 16.11 ) | 7.66 ( 40.39 ) | 7.64 ( 4.1 ) | 2.93 ( 1.91 ) | 0.10% | 0 | None | 2 | low priority |
| Gastroenteritis Viral | 6 | 2.73 ( 1.23 - 6.08 ) | 2.73 ( 6.56 ) | 2.73 ( 1.39 ) | 1.45 ( 0.35 ) | 0.08% | 0 | None | 2 | low priority |
| Performance Status Decreased | 6 | 14.55 ( 6.52 - 32.49 ) | 14.54 ( 75.13 ) | 14.45 ( 7.38 ) | 3.85 ( 2.76 ) | 0.08% | 0 | None | 2 | low priority |
| Swollen Tongue | 6 | 2.45 ( 1.1 - 5.46 ) | 2.45 ( 5.15 ) | 2.45 ( 1.25 ) | 1.29 ( 0.2 ) | 0.08% | 0 | None | 2 | low priority |
| Joint Injury | 6 | 2.27 ( 1.02 - 5.05 ) | 2.27 ( 4.24 ) | 2.27 ( 1.16 ) | 1.18 ( 0.09 ) | 0.08% | 0 | None | 2 | low priority |
| Antineutrophil Cytoplasmic Antibody Increased | 6 | 328.58 ( 138.86 - 777.51 ) | 328.3 ( 1690.86 ) | 283.67 ( 137.98 ) | 8.15 ( 6.98 ) | 0.08% | 0 | None | 2 | low priority |
| Chemotherapy | 6 | 12.73 ( 5.7 - 28.41 ) | 12.72 ( 64.39 ) | 12.65 ( 6.46 ) | 3.66 ( 2.57 ) | 0.08% | 0 | None | 2 | low priority |
| Blood Urine Present | 5 | 2.92 ( 1.21 - 7.02 ) | 2.92 ( 6.3 ) | 2.92 ( 1.4 ) | 1.54 ( 0.36 ) | 0.07% | 0 | None | 2 | low priority |
| Hypervolaemia | 5 | 3.7 ( 1.54 - 8.91 ) | 3.7 ( 9.84 ) | 3.7 ( 1.77 ) | 1.89 ( 0.71 ) | 0.07% | 0 | None | 2 | low priority |
| Cardiac Operation | 5 | 5.24 ( 2.18 - 12.61 ) | 5.24 ( 17.1 ) | 5.23 ( 2.51 ) | 2.39 ( 1.21 ) | 0.07% | 0 | None | 2 | low priority |
| Adverse Reaction | 5 | 3.98 ( 1.65 - 9.57 ) | 3.98 ( 11.13 ) | 3.97 ( 1.91 ) | 1.99 ( 0.81 ) | 0.07% | 0 | None | 2 | low priority |
| Urine Abnormality | 4 | 6.85 ( 2.57 - 18.28 ) | 6.85 ( 19.9 ) | 6.83 ( 3 ) | 2.77 ( 1.48 ) | 0.06% | 0 | None | 2 | low priority |
| Renal Transplant | 4 | 11.45 ( 4.28 - 30.59 ) | 11.44 ( 37.9 ) | 11.38 ( 5 ) | 3.51 ( 2.21 ) | 0.06% | 0 | None | 2 | low priority |
| Inflammatory Marker Increased | 4 | 4.9 ( 1.84 - 13.08 ) | 4.9 ( 12.38 ) | 4.89 ( 2.15 ) | 2.29 ( 1 ) | 0.06% | 0 | None | 2 | low priority |
| Feeling Jittery | 4 | 2.95 ( 1.11 - 7.88 ) | 2.95 ( 5.16 ) | 2.95 ( 1.3 ) | 1.56 ( 0.27 ) | 0.06% | 0 | None | 2 | low priority |
| Oesophageal Disorder | 4 | 13.6 ( 5.09 - 36.35 ) | 13.59 ( 46.35 ) | 13.51 ( 5.93 ) | 3.76 ( 2.46 ) | 0.06% | 0 | None | 2 | low priority |
| Emergency Care | 4 | 6.54 ( 2.45 - 17.46 ) | 6.54 ( 18.71 ) | 6.52 ( 2.87 ) | 2.71 ( 1.41 ) | 0.06% | 0 | None | 2 | low priority |
| Ulcer Haemorrhage | 4 | 7.06 ( 2.65 - 18.86 ) | 7.06 ( 20.74 ) | 7.04 ( 3.1 ) | 2.82 ( 1.52 ) | 0.06% | 0 | None | 2 | low priority |
| Therapy Change | 4 | 2.99 ( 1.12 - 7.96 ) | 2.98 ( 5.27 ) | 2.98 ( 1.31 ) | 1.58 ( 0.28 ) | 0.06% | 0 | None | 2 | low priority |
| Ear Disorder | 3 | 5.68 ( 1.83 - 17.65 ) | 5.68 ( 11.54 ) | 5.67 ( 2.2 ) | 2.5 ( 1.06 ) | 0.04% | 0 | None | 2 | low priority |
| Hyperaesthesia Teeth | 3 | 10.87 ( 3.49 - 33.82 ) | 10.87 ( 26.74 ) | 10.82 ( 4.18 ) | 3.44 ( 1.99 ) | 0.04% | 0 | None | 2 | low priority |
| Parosmia | 3 | 3.94 ( 1.27 - 12.25 ) | 3.94 ( 6.58 ) | 3.94 ( 1.53 ) | 1.98 ( 0.53 ) | 0.04% | 0 | None | 2 | low priority |
| Product Size Issue | 3 | 4.59 ( 1.48 - 14.26 ) | 4.59 ( 8.41 ) | 4.58 ( 1.78 ) | 2.2 ( 0.75 ) | 0.04% | 0 | None | 2 | low priority |
| Escherichia Urinary Tract Infection | 3 | 6.9 ( 2.22 - 21.45 ) | 6.9 ( 15.09 ) | 6.88 ( 2.66 ) | 2.78 ( 1.34 ) | 0.04% | 0 | None | 2 | low priority |
| Serum Ferritin Increased | 3 | 5.76 ( 1.85 - 17.9 ) | 5.76 ( 11.77 ) | 5.75 ( 2.23 ) | 2.52 ( 1.08 ) | 0.04% | 0 | None | 2 | low priority |
| Endotracheal Intubation | 3 | 8.94 ( 2.88 - 27.79 ) | 8.94 ( 21.06 ) | 8.9 ( 3.45 ) | 3.15 ( 1.71 ) | 0.04% | 0 | None | 2 | low priority |
| Blood Creatine Increased | 3 | 5.99 ( 1.93 - 18.62 ) | 5.99 ( 12.44 ) | 5.98 ( 2.32 ) | 2.58 ( 1.13 ) | 0.04% | 0 | None | 2 | low priority |
| Thyroidectomy | 3 | 15.15 ( 4.86 - 47.17 ) | 15.14 ( 39.33 ) | 15.04 ( 5.81 ) | 3.91 ( 2.46 ) | 0.04% | 0 | None | 2 | low priority |
| Blood Blister | 2 | 7.13 ( 1.78 - 28.6 ) | 7.13 ( 10.51 ) | 7.11 ( 2.23 ) | 2.83 ( 1.16 ) | 0.03% | 100.00% | None | 2 | low priority |
| Eyelid Disorder | 2 | 8.83 ( 2.2 - 35.42 ) | 8.83 ( 13.83 ) | 8.8 ( 2.75 ) | 3.14 ( 1.47 ) | 0.03% | 100.00% | None | 2 | low priority |
| Gallbladder Enlargement | 2 | 16.84 ( 4.19 - 67.73 ) | 16.84 ( 29.55 ) | 16.71 ( 5.21 ) | 4.06 ( 2.39 ) | 0.03% | 50.00% | IME | 2 | low priority |
| Pneumonia Legionella | 2 | 13.04 ( 3.25 - 52.38 ) | 13.04 ( 22.09 ) | 12.96 ( 4.05 ) | 3.7 ( 2.02 ) | 0.03% | 50.00% | IME | 2 | low priority |
| Pneumomediastinum | 2 | 8.59 ( 2.14 - 34.47 ) | 8.59 ( 13.36 ) | 8.56 ( 2.68 ) | 3.1 ( 1.43 ) | 0.03% | 50.00% | IME | 2 | low priority |
| Klebsiella Bacteraemia | 2 | 14.91 ( 3.71 - 59.92 ) | 14.91 ( 25.76 ) | 14.81 ( 4.62 ) | 3.89 ( 2.21 ) | 0.03% | 50.00% | IME | 2 | low priority |
| Emphysematous Cystitis | 2 | 50.73 ( 12.47 - 206.31 ) | 50.71 ( 95.15 ) | 49.53 ( 15.31 ) | 5.63 ( 3.93 ) | 0.03% | 50.00% | IME | 2 | low priority |
| Pneumonia Staphylococcal | 2 | 12.57 ( 3.13 - 50.47 ) | 12.56 ( 21.16 ) | 12.49 ( 3.9 ) | 3.64 ( 1.97 ) | 0.03% | 50.00% | IME | 2 | low priority |
| Legionella Infection | 2 | 37.81 ( 9.34 - 153.14 ) | 37.8 ( 70.38 ) | 37.15 ( 11.53 ) | 5.22 ( 3.53 ) | 0.03% | 50.00% | IME | 2 | low priority |
| Vomiting | 70 | 1.5 ( 1.18 - 1.89 ) | 1.49 ( 11.38 ) | 1.49 ( 1.22 ) | 0.58 ( 0.23 ) | 0.98% | 2.86% | None | 1 | low priority |
| Insomnia | 47 | 1.91 ( 1.43 - 2.55 ) | 1.91 ( 20.26 ) | 1.9 ( 1.5 ) | 0.93 ( 0.51 ) | 0.66% | 0 | None | 1 | low priority |
| Decreased Appetite | 44 | 1.61 ( 1.2 - 2.17 ) | 1.61 ( 10.19 ) | 1.61 ( 1.26 ) | 0.69 ( 0.25 ) | 0.62% | 4.55% | None | 1 | low priority |
| Urinary Tract Infection | 38 | 1.84 ( 1.34 - 2.53 ) | 1.84 ( 14.55 ) | 1.84 ( 1.41 ) | 0.88 ( 0.41 ) | 0.53% | 5.26% | None | 1 | low priority |
| Blood Pressure Increased | 35 | 1.94 ( 1.39 - 2.7 ) | 1.93 ( 15.74 ) | 1.93 ( 1.46 ) | 0.95 ( 0.47 ) | 0.49% | 2.86% | None | 1 | low priority |
| Paraesthesia | 24 | 1.51 ( 1.01 - 2.25 ) | 1.5 ( 4.06 ) | 1.5 ( 1.08 ) | 0.59 ( 0.01 ) | 0.34% | 0 | None | 1 | low priority |
| Influenza | 22 | 1.66 ( 1.09 - 2.52 ) | 1.66 ( 5.76 ) | 1.66 ( 1.17 ) | 0.73 ( 0.13 ) | 0.31% | 4.55% | None | 1 | low priority |
| Abdominal Distension | 20 | 1.86 ( 1.2 - 2.88 ) | 1.86 ( 7.91 ) | 1.86 ( 1.29 ) | 0.89 ( 0.26 ) | 0.28% | 5.00% | None | 1 | low priority |
| Oropharyngeal Pain | 19 | 1.63 ( 1.04 - 2.56 ) | 1.63 ( 4.63 ) | 1.63 ( 1.12 ) | 0.7 ( 0.06 ) | 0.27% | 10.53% | None | 1 | low priority |
| Herpes Zoster | 13 | 1.95 ( 1.13 - 3.36 ) | 1.95 ( 5.98 ) | 1.94 ( 1.23 ) | 0.96 ( 0.19 ) | 0.18% | 0 | None | 1 | low priority |
| Respiration Abnormal | 3 | 3.04 ( 0.98 - 9.44 ) | 3.04 ( 4.1 ) | 3.04 ( 1.18 ) | 1.6 ( 0.16 ) | 0.04% | 0 | None | 1 | low priority |
| Hyperbilirubinaemia | 3 | 2.85 ( 0.92 - 8.86 ) | 2.85 ( 3.61 ) | 2.85 ( 1.11 ) | 1.51 ( 0.07 ) | 0.04% | 0 | IME | 1 | low priority |
| Thoracic Vertebral Fracture | 2 | 6.6 ( 1.65 - 26.46 ) | 6.6 ( 9.47 ) | 6.58 ( 2.06 ) | 2.72 ( 1.05 ) | 0.03% | 0 | IME | 1 | low priority |
| Cardiorenal Syndrome | 2 | 27.19 ( 6.74 - 109.72 ) | 27.18 ( 49.78 ) | 26.84 ( 8.35 ) | 4.75 ( 3.06 ) | 0.03% | 0 | IME | 1 | low priority |
| Atypical Mycobacterial Infection | 2 | 10.17 ( 2.53 - 40.81 ) | 10.17 ( 16.45 ) | 10.12 ( 3.16 ) | 3.34 ( 1.67 ) | 0.03% | 0 | IME | 1 | low priority |
| Spinal Artery Thrombosis | 2 | 693.28 ( 139.9 - 3435.54 ) | 693.08 ( 1036.63 ) | 520.06 ( 136.29 ) | 9.02 ( 7.1 ) | 0.03% | 0 | IME | 1 | low priority |
| Muscle Abscess | 2 | 25.36 ( 6.29 - 102.29 ) | 25.36 ( 46.23 ) | 25.06 ( 7.8 ) | 4.65 ( 2.97 ) | 0.03% | 0 | IME | 1 | low priority |
| Jc Virus Infection | 2 | 9.33 ( 2.32 - 37.41 ) | 9.32 ( 14.8 ) | 9.29 ( 2.9 ) | 3.22 ( 1.54 ) | 0.03% | 0 | IME | 1 | low priority |
| Glomerulonephritis | 2 | 7.25 ( 1.81 - 29.05 ) | 7.24 ( 10.73 ) | 7.22 ( 2.26 ) | 2.85 ( 1.18 ) | 0.03% | 0 | IME | 1 | low priority |
| Oedema | 10 | 1.85 ( 0.99 - 3.44 ) | 1.85 ( 3.88 ) | 1.85 ( 1.1 ) | 0.88 ( 0.01 ) | 0.14% | 0 | None | 0 | low priority |
| Paraesthesia Oral | 4 | 2.56 ( 0.96 - 6.82 ) | 2.56 ( 3.78 ) | 2.55 ( 1.12 ) | 1.35 ( 0.06 ) | 0.06% | 0 | None | 0 | low priority |
| Immune System Disorder | 4 | 2.46 ( 0.92 - 6.56 ) | 2.46 ( 3.46 ) | 2.46 ( 1.08 ) | 1.3 ( 0.01 ) | 0.06% | 0 | None | 0 | low priority |
| Auditory Disorder | 2 | 9.39 ( 2.34 - 37.67 ) | 9.39 ( 14.92 ) | 9.35 ( 2.92 ) | 3.22 ( 1.55 ) | 0.03% | 0 | None | 0 | low priority |
| Seborrhoeic Dermatitis | 2 | 8.93 ( 2.23 - 35.8 ) | 8.92 ( 14.01 ) | 8.89 ( 2.78 ) | 3.15 ( 1.48 ) | 0.03% | 0 | None | 0 | low priority |
| Impulsive Behaviour | 2 | 7.39 ( 1.84 - 29.62 ) | 7.39 ( 11 ) | 7.36 ( 2.3 ) | 2.88 ( 1.21 ) | 0.03% | 0 | None | 0 | low priority |
| Latent Tuberculosis | 2 | 7.04 ( 1.76 - 28.21 ) | 7.04 ( 10.32 ) | 7.02 ( 2.2 ) | 2.81 ( 1.14 ) | 0.03% | 0 | None | 0 | low priority |
| Pharyngeal Erythema | 2 | 9.67 ( 2.41 - 38.81 ) | 9.67 ( 15.48 ) | 9.63 ( 3.01 ) | 3.27 ( 1.6 ) | 0.03% | 0 | None | 0 | low priority |
| Trichorrhexis | 2 | 7.98 ( 1.99 - 32.01 ) | 7.98 ( 12.17 ) | 7.96 ( 2.49 ) | 2.99 ( 1.32 ) | 0.03% | 0 | None | 0 | low priority |
| Nasal Disorder | 2 | 6.55 ( 1.63 - 26.25 ) | 6.55 ( 9.37 ) | 6.53 ( 2.04 ) | 2.71 ( 1.04 ) | 0.03% | 0 | None | 0 | low priority |
| Oxygen Therapy | 2 | 6.53 ( 1.63 - 26.17 ) | 6.53 ( 9.33 ) | 6.51 ( 2.04 ) | 2.7 ( 1.03 ) | 0.03% | 0 | None | 0 | low priority |
| Eye Symptom | 2 | 29.09 ( 7.2 - 117.46 ) | 29.08 ( 53.48 ) | 28.69 ( 8.92 ) | 4.84 ( 3.16 ) | 0.03% | 0 | None | 0 | low priority |
| Protein Total Increased | 2 | 7.01 ( 1.75 - 28.12 ) | 7.01 ( 10.28 ) | 6.99 ( 2.19 ) | 2.81 ( 1.14 ) | 0.03% | 0 | None | 0 | low priority |
| Intensive Care | 2 | 8.06 ( 2.01 - 32.33 ) | 8.06 ( 12.32 ) | 8.03 ( 2.51 ) | 3.01 ( 1.33 ) | 0.03% | 0 | None | 0 | low priority |

Caption: DME designated medical event, IME important medical event.
